# *Ex vivo* expansion of alveolar macrophages with *Mycobacterium tuberculosis* from the resected lungs of patients with pulmonary tuberculosis

**DOI:** 10.1371/journal.pone.0191918

**Published:** 2018-02-05

**Authors:** Elena Ufimtseva, Natalya Eremeeva, Ekaterina Petrunina, Tatiana Umpeleva, Svetlana Karskanova, Sergey Bayborodin, Diana Vakhrusheva, Marionella Kravchenko, Sergey Skornyakov

**Affiliations:** 1 Laboratory of Medical Biotechnology, Research Institute of Biochemistry, Novosibirsk, Russia; 2 Scientific Department, Ural Research Institute for Phthisiopulmonology, National Medical Research Center of Tuberculosis and Infectious Diseases of Ministry of Health of the Russian Federation, Yekaterinburg, Russia; 3 Shared Center for Microscopic Analysis of Biological Objects, Federal Research Center Institute of Cytology and Genetics, Novosibirsk, Russia; Rutgers Biomedical and Health Sciences, UNITED STATES

## Abstract

Tuberculosis (TB), with the *Mycobacterium tuberculosis* (*Mtb*) as the causative agent, remains to be a serious world health problem. Traditional methods used for the study of *Mtb* in the lungs of TB patients do not provide information about the number and functional status of *Mtb*, especially if *Mtb* are located in alveolar macrophages. We have developed a technique to produce *ex vivo* cultures of cells from different parts of lung tissues surgically removed from patients with pulmonary TB and compared data on the number of cells with *Mtb* inferred by the proposed technique to the results of bacteriological and histological analyses used for examination of the resected lungs. The *ex vivo* cultures of cells obtained from the resected lungs of all patients were largely composed of CD14-positive alveolar macrophages, foamy or not, with or without *Mtb*. Lymphocytes, fibroblasts, neutrophils, and multinucleate Langhans giant cells were also observed. We found alveolar macrophages with *Mtb* in the *ex vivo* cultures of cells from the resected lungs of even those TB patients, whose sputum smears and lung tissues did not contain acid-fast *Mtb* or reveal growing *Mtb* colonies on dense medium. The detection of alveolar macrophages with *Mtb* in *ex vivo* culture as soon as 16–18 h after isolation of cells from the resected lungs of all TB patients suggests that the technique proposed for assessing the level of infection in alveolar macrophages of TB patients has higher sensitivity than do prolonged bacteriological or pathomorphological methods. The proposed technique allowed us to rapidly (in two days after surgery) determine the level of infection with *Mtb* in the cells of the resected lungs of TB patients and, by the presence or absence of *Mtb* colonies, including those with cording morphology, the functional status of the TB agent at the time of surgery.

## Introduction

Tuberculosis, with the *M*. *tuberculosis* as the causative agent, accounts for about 2 million deaths annually and is one of the leading causes of deaths from infectious disease caused by a single agent [1]. According to WHO reports, one-third of the world's human population is infected with *Mtb* and each infected with this bacterium has a 5–10% risk of developing active TB, which amounts to 8–9 million new cases annually [1, 2]. The pathogenesis of TB depends on the intracellular persistence of *Mtb* in host macrophages [3, 4]. Macrophages are the cells of the innate immunity system. They are part of the primary immune response, which acts to attack and kill phagocytosed infectious agents, including *Mtb*. However, in some macrophages *Mtb* survive and replicate [2–6]. It is considered that the pivotal phase of TB pathogenesis in humans is granulomatous alterations in tissue structure—that of lung tissue in the first place—around foci of infection [2, 7, 8]. A TB granuloma is a highly organized chronic inflammatory structure with a complex cellular composition and many biochemical reactions running in it, which occurs in the form of a local aggregation of mononuclear cells, mostly macrophages, with *Mtb* in them [8–11]. Additionally, granulomatous TB lesions are observed to contain dendritic cells, lymphocytes, neutrophils, fibroblasts, and multinucleate Langhans giant cells [8, 9, 11, 12]. Granulomas, on the one hand, restrict dissemination of TB infection, while, on the other hand, provide for latency and set the stage for reactivation [13, 14]. The organism of any individual with pulmonary TB displays a broad spectrum of physiologically distinct TB lesions with a wide range of pathological, microbiological, and immunological features [8, 9, 13, 15, 16]. As heterogeneous as they are, many of the TB lesions in the lungs of humans infected with *Mtb* appear as enclosed structures with caseous necrosis in the center surrounded first by macrophages and finally by peripheral fibrosis [8, 9, 15, 16]. Mycobacteria in the caseous necrotic centers of these granulomas do not normally replicate, because they are stressed by hypoxia, nutrient deprivation and an acid environment [8, 13]. However, as the TB process progresses, caseous necrotic foci in the lungs may liquefy, giving rise to cavitation and allowing caseous matter with *Mtb* to reach the bronchi [8]. It has been established that *Mtb* replicating outside and probably within cells occur in large numbers in well-aerated cavity walls [8, 15, 17]. As a result, TB patients cough out sputum from their bronchi and transmit *Mtb* to people nearby.

Anti-TB chemotherapies are to eliminate *Mtb* both actively replicating in the cavity walls and present in caseous necrotic centers as scanty, probably dormant bacteria [2, 8, 13, 14]. However, the emergence and propagation of multidrug-resistant (MDR) and extensively drug-resistant (XDR) *Mtb* have substantially reduced the efficiency of the existing anti-TB chemotherapies and triggered the search for new ways of TB prevention and treatment [1, 18, 19]. One of the effective approaches used in TB treatment is by surgical removal of the affected lung part [2, 13, 17, 20]. Resected lung parts represent a valuable source of information for specifying the diagnosis and the biological features of the TB agent, which is extremely important for assessing the efficiency of preoperative treatment and choosing a postoperative treatment strategy [9, 17, 20]. This is especially important when nothing was known about the properties of the pathogen from respiratory material before surgery [20, 21].

To assess the level of infection by *Mtb* in the lung tissues of TB patients in clinical settings, the bacteriological method is used, which is by seeding tissue homogenates of resected organs onto special media and counting colony-forming units [2]. However, this method provides only general information about the number of *Mtb* in the material, telling nothing about how many cells are infected or how many microbes each of those infected cells contains–nor do they tell about the functional status of the TB agent in them. Furthermore, this method is very slow: because of low replication rates of *Mtb*, it takes 1–2 months after seeding to make final inferences about the infection level in lung tissues and the specific properties of the pathogen. In the human organism, *Mtb* occurs either in host cells or in necrotic tissue, that is, in the environments that are not like well-aerated liquid or solid nutrient media. Additionally, the TB agent in the human organism is under the control of the immune system permanently and, therefore, the status of *Mtb* in an organism may be distinct from that observed *in vitro*.

With histological methods, it is extremely difficult to detect *Mtb* in alveolar macrophages on tissue sections from resected lung parts, whether by staining for acid-fast *Mtb* [15–17] or by *in situ* detection of the transcripts of various *Mtb* genes [22]. Therefore, histological methods are of little help in assessing infection levels in macrophages in the study organ. Histology of excised lung tissue from TB patients is basically aimed at specifying the diagnosis and the pathomorphological picture of the disease [2, 9, 17, 20].

At present, TB is diagnosed using molecular genetic methods, quite rapid and highly sensitive [20, 23]. However, sole detection of *Mtb* DNA in the study material does not tell about exact levels of infection by *Mtb* in the lung macrophages–nor does it tell about the viability or metabolic status of the TB agent. As is known, mycobacterial DNA can persist in human lung tissues for a long time after the pathogen has been destroyed [24, 25].

Because the cells hosting pathogenic *Mtb* are largely leukocytes, attempts have been made to obtain them from TB patients’ organisms. Ulrichs and the co-workers [9] obtained mononuclear cells from different tissue types in resected lungs using collagenase and Ficoll gradient centrifugation, which allowed these authors to assess only the relative number of macrophages and lymphocytes present in the study piece of lung tissue. Isolation of cells, largely neutrophils, from the bronchoalveolar lavage (BAL) fluid, sputum, and cavity caseum of patients with pulmonary TB allowed the number of cells infected with *Mtb* to be assessed [26]. In another study [27], Fu and the co-workers did not obtain viable cells from sputa, but caseous matter, which is typical of the sputum coughed out by TB patients; nor could they estimate–not even roughly–the *Mtb* infection levels in the host cells. Thus, even though the control of the population of alveolar macrophages infected with *Mtb* in the TB patients’ lungs is important, the issue remains poorly explored. Therefore, to assess the efficiency of the therapy being given, to see when a personalized revision of treatment regimens is needed and to be able to make predictions as to how TB infection will proceed in post-operative TB patients, new techniques are required that enable rapid detection and analysis of cells with *Mtb* in TB patients [28].

We started our search for ways to analyze *Mtb* infection levels in macrophages in patients with pulmonary TB by using a mouse model of latent TB infection and *ex vivo* monolayer cultures of cells that had migrated from individual granulomas obtained from the spleens, lungs, and bone marrow of mice infected with the Bacillus Calmette-Guérin (BCG) vaccine [11, 29]. The BALB/c mice used had, at all times of latent TB infection, differences in the number of granulomas with BCG-containing cells as well as in the number of cells containing BCG mycobacteria in the granulomas. Not a single mouse granuloma–not even the smallest–was found to have all macrophages infected [29]. Granuloma macrophages and dendritic cells were for the first time observed to contain colonies of BCG mycobacteria with cording morphology, which correlates with the virulence of the mycobacteria [29]. A comparison made between BCG mycobacteria in *ex vivo* cultures of mouse granuloma macrophages and in macrophages infected with the BCG vaccine *in vitro* in the cell cultures of bone marrow and peritoneal exudate from intact mice revealed that host cells and mycobacteria themselves have different, even opposing, responses to infection in different model systems [30].

Based on our previous work on the isolation of cells from mouse granulomatous inflammatory structures and proceeding with these cells in *ex vivo* culture, we have developed a technique to isolate alveolar macrophages from the resected lung tissues of patients with pulmonary TB for rapid quantification of *Mtb* infection levels in macrophages [31]. The results, obtained using the proposed technique, have decision-making potential when questions arise regarding the application and revision of prophylactic and medical treatment measures against pulmonary TB in humans.

## Materials and methods

### Study design

A technique is reported for obtaining alveolar macrophages from lung parts resected during elective surgery from adults with pulmonary TB with the aim of a rapid assessment of the level of infection with *M*. *tuberculosis* in the cells from post-operative patients. Data on the number of alveolar macrophages inferred in two days using the proposed technique were compared to the results of prolonged bacteriological and histological analyses used in routine clinical practice for examination of lung tissue resected from TB in-patients. The participants were selected on a random basis during several months. All the patients’ characteristics, procedures and experiments are presented and discussed in tables, figures, and legends to them.

### Patients and ethics statement

Lung tissue specimens were obtained from 21 patients with pulmonary TB at the Department of Thoracic Surgery of the National Medical Research Center of Tuberculosis and Infectious Diseases (Yekaterinburg, Russia) over the period from August 2014 to May 2015. Standard preoperative procedures, including diagnosis of mycobacteria in sputum, chest radiography, and Computed Tomography were performed for all patients (S1 and S2 Tables). All patients included in the present study had been referred for the surgical management of treatment-refractory pulmonary TB. Destroyed regions were removed within the anatomical borders of lung parts from all patients with clinically active TB. All patients were residents of the Ural province of Russia and had received TB treatment supervised by their local clinics. Preoperative *M*. *tuberculosis* definition from the diagnostic sputa and chest radiographs were performed on all patients (S1 Table). All procedures involving patients were fully reviewed and approved by the Ethics Committee of the National Medical Research Center of Tuberculosis and Infectious Diseases (27/2014/07/02) and conducted in accordance with the principles expressed in the Helsinki Declaration. Written informed consent was obtained from each patient in this study.

### Lung tissue processing

Immediately after surgery, removed lung tissue from each patient was transferred to a biological safety level 3 facility for pathological examination and dissection. In brief, TB lesions were identified macroscopically. For DNA analysis, ~0.5 g of caseous matter from a necrotizing TB lesion was homogenized using a sterile pestle in 1.5-ml tubes with inactivating reagent A (Syntol, Russia) and incubated for 30 min. ~2 g of lung tissue with TB lesions was homogenized and cultured on Lowenstein-Jensen (LJ) medium (Himedia Laboratories, India). Pieces of lung tissues (~10–30 g) obtained from lung parts distant from macroscopic TB lesions and cavities were collected for producing alveolar macrophages for each patient (*n* = 20), but only the cavity wall of the resected lung was collected for patient 6. The remainders of the lungs were immersed in 10% paraformaldehyde in phosphate-buffered saline (PBS, pH 7.4) for histological analysis of TB lesions.

### Isolation and *ex vivo* culture of alveolar macrophages

Alveolar macrophages were obtained from the specimens of surgically resected lung tissue as described in [31, 32]. In brief, lung tissue specimens were cut into small pieces and incubated in PBS. For separating cell suspension containing alveolar macrophages from closed granulomatous fibrotic tissue, the lung pieces were further rubbed through the metal screen of a sieve with pores 0.5–2.0 mm diameter in PBS. Cells were isolated from the organ homogenate by centrifugation at 400 *g* for 5 min at room temperature and washed once in PBS. Cell pellets in a complete growth RPMI 1640 medium containing 10% fetal bovine serum, 2 mM glutamine and 50 μg/ml gentamicin (BioloT, St. Petersburg, Russia) were placed to 24-well plates (Orange Scientific, Belgium) with cover glasses in the bottom and cultured in 0.5 ml complete growth medium for 16–18 hours at +37 °C in an atmosphere containing 5% CO_2_.

### Cell staining

At hours 16–18 of *ex vivo* culture after removal of growth medium with dead cell debris, monolayer cultures of cells on cover slips were washed twice with PBS for removal of nonadherent cells and then fixed with 4% formaldehyde solution in PBS for 10 min at room temperature. After washing with PBS, the viability of cells on the cover slips was, as checked by trypan blue staining, 100%. To visualize acid-fast mycobacteria within host cells, some preparations were washed with PBS and stained by the Ziehl-Neelsen (ZN) method. The cells after ZN staining were further counterstained with Mayer's hematoxylin.

In the experiments using Nile red (Invitrogen, USA, N1142), the cell preparations were incubated with 10 μM of the dye for 15 min at +37 °C in 5% CO_2_ before fixation. The cell preparations were fixed as described above, washed with PBS, blocked in PBS solution containing 2% BSA, and finally incubated first with rabbit polyclonal primary antibodies to *Mycobacteria* LAM (Abcam, England, ab20832) diluted 1:200, then with Alexa 488-conjugated goat anti-rabbit IgG secondary antibodies (Thermo Fisher Scientific, USA, A11034) diluted 1:400. Some of the fixed cell preparations were washed with PBS, treated within 2 min in 0.3% Triton-X100 solution, blocked in PBS solution containing 2% BSA, and incubated with rabbit monoclonal primary antibodies to human CD14 (Spring Bioscience, USA, M492) and mouse monoclonal primary antibodies to *M*. *tuberculosis* 38kDa (Abcam, England, ab183165) diluted 1:100 and 1:1000, respectively. Fluorescent visualization of CD14 and mycobacterial 38-kDa antigen was enabled using secondary goat polyclonal Alexa 488-conjugated secondary antibodies to rabbit IgG (Thermo Fisher Scientific, USA, A11034) and Alexa 555-conjugated secondary antibodies to mouse IgG (Invitrogen, USA, A21422) diluted 1:400 and 1:500, respectively. The cell preparations were incubated with the appropriate antibodies for 60 min at room temperature. Fluorescent staining was analyzed using the VECTASHIELD^®^ Mounting Medium with DAPI (4´,6-diamidino-2-phenylindole) (Vector Laboratories, USA, H-1200).

### Histology

The parts of the lungs from each patient were fixed in 10% paraformaldehyde in PBS overnight and embedded in paraffin using an STP 120–3 spinning tissue processor (Zeiss), sectioned at a 4-μm thickness and stained with hematoxylin and eosin or a mixture of picric acid and fuchsin acid to detect collagen and other types of connective tissue or by the ZN method to visualize acid-fast mycobacteria.

### Microscopy

The cytological and histological preparations were examined at the Shared Center for Microscopic Analysis of Biological Objects of the Institute of Cytology and Genetics, SB RAS, using an Axioscop 2 *plus* microscope (Zeiss) and objectives with various magnifications (Zeiss), and photographed using an AxioCam HRc camera (Zeiss); the images were analyzed using the AxioVision 4.7 microscopy software (Zeiss). Cell preparations stained with fluorescent dyes were examined under an LSM 510 or an LSM 780 laser scanning confocal microscope (Zeiss) using the LSM Image Browser and ZEN 2010 software (Zeiss). All cells were counted separately on each cover slip for each patient in each test.

### PCR analysis

After incubation with inactivating reagent A (Syntol, Russia), samples were centrifuged for 5 min at 13,000 rpm. The precipitate was used for DNA extraction with M-Sorb-Tub reagent kits (Syntol, Russia). The DNA obtained was used for PCR with AmpliTub-RV reagent kits (Syntol, Russia). This kit detects specific DNA fragments of the *IS6110* gene, which is present in multiple copies in most *M*. *tuberculosis* complex strains, and determines the amount of *regX*, a specific DNA fragment, which is represented by a single copy in the *M*. *tuberculosis* complex genome. PCR was performed using a CFX96 analyzer (Bio-Rad, USA).

### Bacteriology

Before surgery, the smears of sputum were collected from each patient and stained by the ZN method and Fluorescent Stains Kit for *Mycobacteria* with auramine and rhodamine (Himedia Laboratories, India). After surgery, decontamination of lung tissue specimens was performed by the standard NaOH-NALC method [33]. After decontamination and homogenization, each specimen was inoculated on LJ medium (Himedia Laboratories, India) for isolation of *Mtb*. The time to positivity of the LJ medium was 1–2 months. All grown *Mtb* cultures were identified and confirmed as being in the *M*. *tuberculosis* complex using standard procedures.

### Statistics

Statistical data processing was performed using MS Excel 2007 (Microsoft). Differences were tested using Student’s *t*-test and considered statistically significant at *P* < 0.05.

## Results

### Isolation of cells from the resected lungs of TB patients

**Fig 1:**
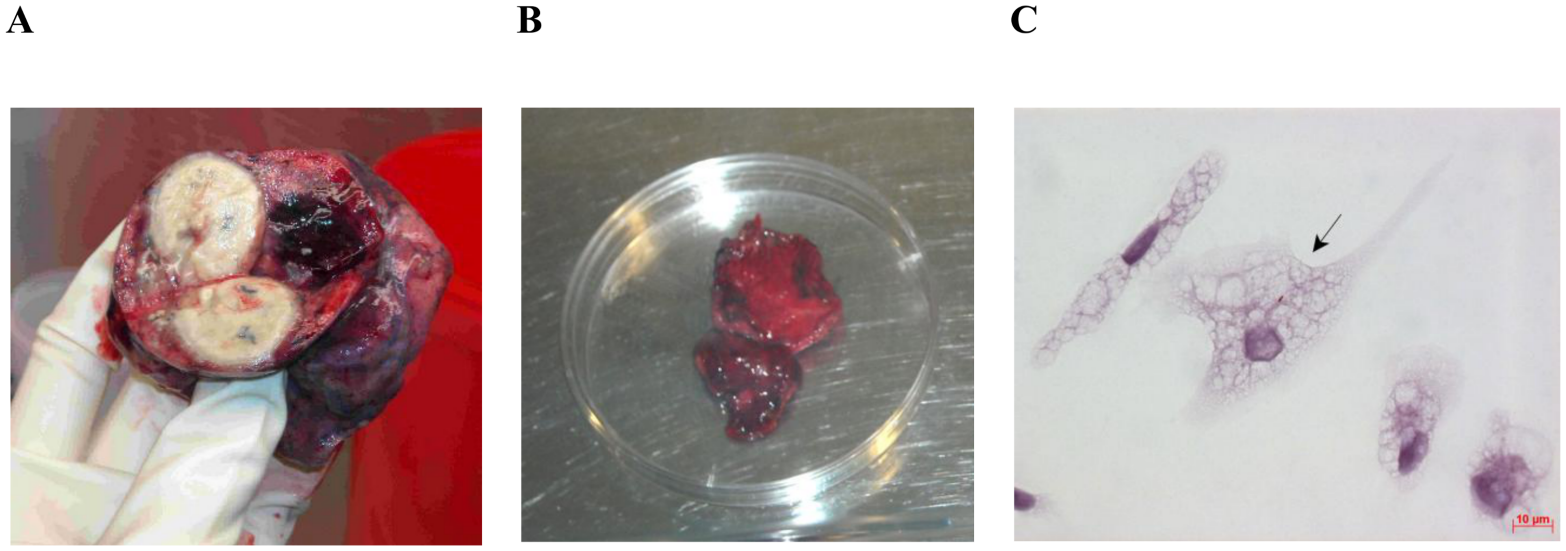
Lung tissue used for *ex vivo* expansion of alveolar macrophages. (A) The upper lobe of the left lung surgically removed from patient 8. (B) The tissue specimen of the part of the resected lung (A) distant from macroscopic lesions. The diameter of the Petri dish is 10 cm. (C) Alveolar macrophages produced from the lung tissue specimen (B) and stained by the ZN method after *ex vivo* culture for 18 hours. The black arrow points to an alveolar macrophage with acid-fast *Mtb*. The scale bar is 10 μm.

Cells were isolated from small specimens of lung tissue resected during elective surgery from 21 patients with pulmonary TB (Fig 1A and 1B, S1A, S1B, S1E, S1F, S1I, S1J, S1M and S1N Fig). The patients referred to surgery are characterized in S1 Table. Patients, from whom affected lung sections were resected, had different clinical symptoms of TB before surgery; however, according to X-ray, all these individuals had fibrotic and caseotic TB lesions in their lungs. Most of the patients (*n* = 16) were diagnosed with MDR TB, and patient 6, with XDR TB. Five patients (6÷10) had cavities in their lungs. Based on clinical manifestations, chest radiography, and Computed Tomography (in S2 Table), all the patients were divided into three severity groups with different extents of TB disease: “advanced” (patients 6, 7, and 10), “moderate” (patients 5, 8, 15, 16, and 20), and “minimal” (the other 13 patients). Sputum smears were examined from all the patients before surgery; however, only material from those with cavities was found to contain acid-fast mycobacteria (except for patient 9).

In all the patients, except for patient 6, lung tissue specimens for cell isolation were obtained from sites distal to cavities or other macroscopic TB foci (Fig 1A and 1B, S1E, S1F, S1I, S1J, S1M and S1N Fig). Cells from patient 6 were obtained only from the cavity wall (S1A and S1B Fig). All the lung specimens used for cell isolation had large amounts of fibrotic tissue with small enclosed TB granulomas in it (Fig 1B, S1B, S1F, S1J and S1N Fig). This granulomatous fibrotic tissue with caseous TB lesions was separated from the cell suspension by homogenization of lung tissues and by washing off cells for *ex vivo* culture (S1C, S1G, S1K and S1O Fig) and then discarded. The pellets obtained by centrifugation of the cell suspension and containing, according to microscopic data, a large number of erythrocytes and small cells with light cytoplasm (probably, lymphocytes) and a much lesser number of larger light cells (probably, macrophages) were seeded to 24-well plates with cover glasses in the bottom separately for each patient.

Thus, as we worked with tissue samples from the resected lungs of TB patients, we separated granulomatous fibrotic tissue with caseous TB lesions from the cells of the surrounding tissue and seeded those cells in *ex vivo* culture.

### Cell composition of *ex vivo* cultures from TB patients’ lungs

**Fig 2:**
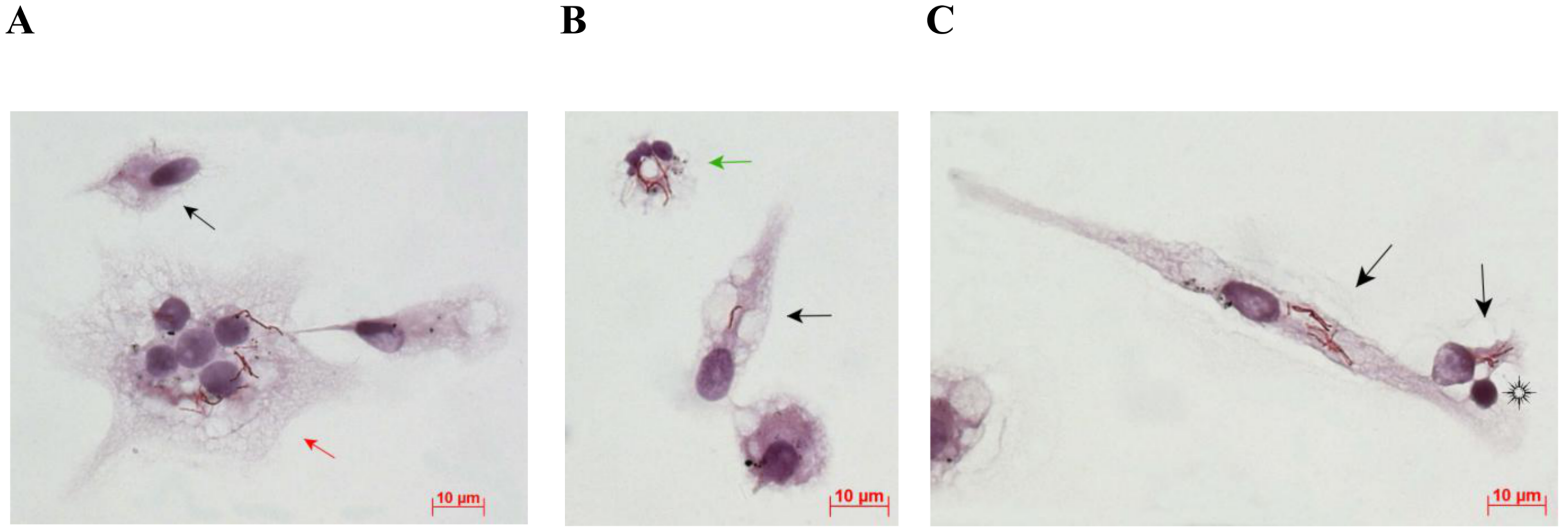
Different cell types obtained from the cavity wall in the resected lung of patient 6 and stained by the ZN method after *ex vivo* culture for 16 hours. (A, B) A Langhans giant cell and a neutrophil containing acid-fast *Mtb* are indicated by the red and green arrows, respectively. (C) A lymphocyte interplaying with alveolar macrophages is indicated by the black snowflake. The other cells (A, B, C) are alveolar macrophages. Those with acid-fast *Mtb* are indicated by the black arrows, others are uninfected. The scale bars are 10 μm each.

**Fig 3:**
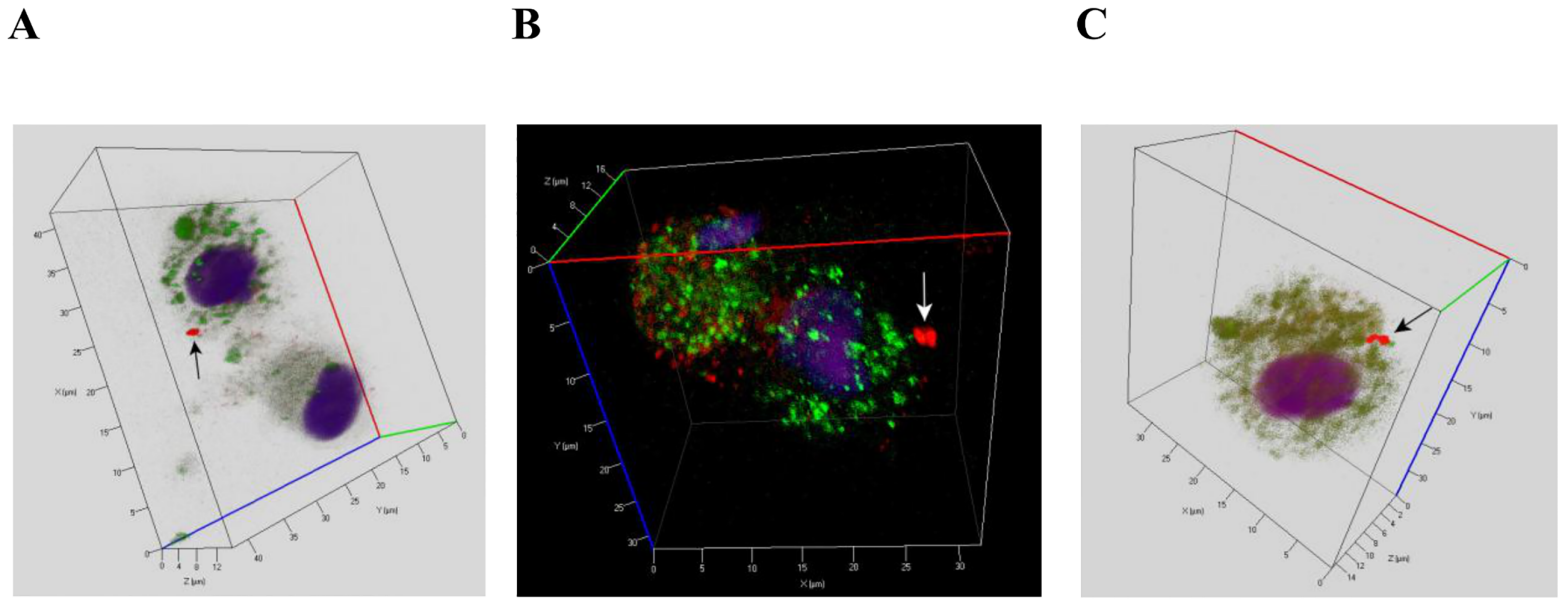
CD14 staining of alveolar macrophages with *Mtb* performed after *ex vivo* culture for 18 hours. (A, B, C) Representative confocal fluorescent 3D images show alveolar macrophages stained by human CD14-specific (green signal) and *Mtb* Ag38-specific antibodies (red signal). Nuclei are stained by DAPI (blue signal). Alveolar macrophages were obtained from the resected lungs of patients 16 (A, B) and 18 (C). Black or white arrows indicate a single *Mtb* (A) and mycobacterial colonies (B, C) residing within alveolar macrophages.

The cell composition of monolayer cultures on cover glasses inferred for each TB patient after *ex vivo* culture for 16–18 hours and removal of nonadherent cells is presented in S3 Table. The *ex vivo* cultures of cells obtained from the cavity wall and distant parts of resected lung tissue were largely composed of alveolar macrophages (90–99%), with or without *Mtb*. All alveolar macrophages had lightly staining vacuolar cytoplasm with various numbers of dense inclusions, which is typical of alveolar macrophages [34], and a large number of cell membrane protrusions (Figs 1C and 2A–2C, S1D, S1H, S1K and S1P Fig). It is noteworthy that no phagosomes with engulfed lymphocytes, erythrocytes, and platelets were observed in alveolar macrophages (Figs 1C and 2A–2C, S1D, S1H, S1K and S1P Fig). Some of the alveolar macrophages obtained from most patients (*n* = 18) had a large number of denser dark inclusions in the cytoplasm and were what is called smokers’ macrophages (S1H Fig). The presence of smokers’ macrophages in the lung tissue was confirmed by histology of the resected lungs of these patients. CD14, the receptor for lipopolysaccharide in bacterial cell walls and the macrophage/monocyte-specific leukocyte marker, occurred at an increased density in all alveolar macrophages, whether with or without *Mtb* (Fig 3A–3C).

In addition to alveolar macrophages, five more cell types were observed: dendritic cells, lymphocytes, fibroblasts, neutrophils, and multinucleate Langhans giant cells. The population sizes of other cell types were much smaller. Lymphocytes were largely located on alveolar macrophages and interacted with them (Fig 2C). Dendritic cells were mostly smaller than macrophages. These cells had an increased number of tiny cell membrane protrusions, and their nuclei and cytoplasm were stained by histochemical dyes very densely. A higher number of polymorphonuclear neutrophils was observed both in the *ex vivo* cell cultures (about 22% of cells) and in the histological sections of resected lung tissues from only patient 10, which is known to be a predictor of a poor prognosis in TB in humans [35, 36]. Multinucleate Langhans giant cells were found in the *ex vivo* cultures of cells from a large number of patients (Fig 2A, S2A–S2D Fig). Cells with more than five nuclei in each were attributed to this cell type. Langhans giant cells containing both 5–7 and up to 20–25 nuclei were observed (S2A–S2D Fig). These cells also differed in the number of tobacco smoke particles they contained (S2B and S2D Fig).

**Fig 4:**
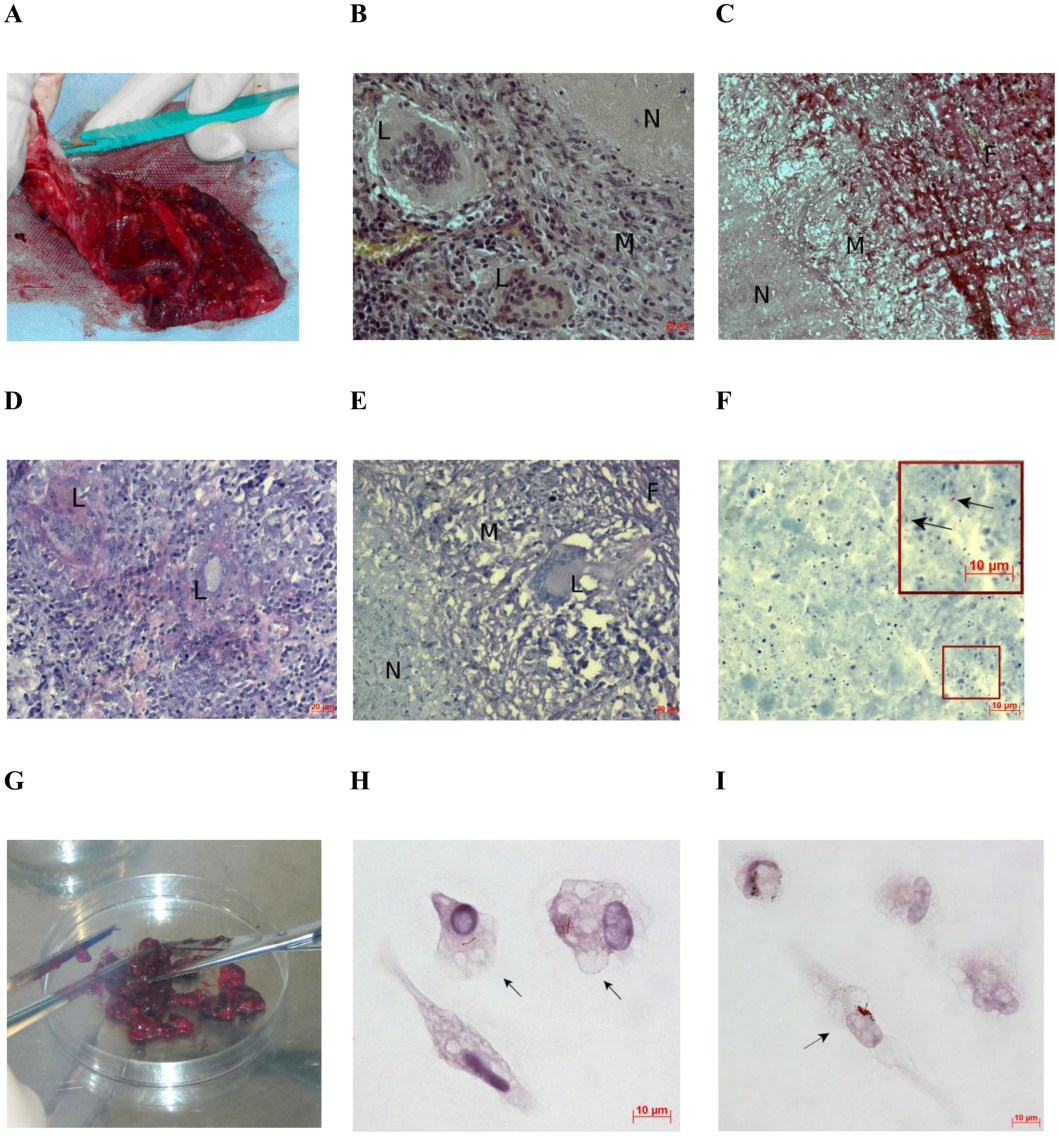
Histological examination of the resected lung from patient 7 shows the absence of cells with *Mtb*. *Ex vivo* analysis of alveolar macrophages disagrees. (A) A surgically removed part of the lung. (B-F) Multiple TB lesions detected by histological analysis of tissue from the resected lung (A): N, caseous necrosis; M, a ring of macrophages; F, fibrous capsule; L, multinucleate Langhans giant cell. The scale bars are (B, D, E) 20 μm each, (C) 50 μm, and (F) 10 μm. (B, C, and E, F) Representative histological images show enclosed necrotizing granulomas stained by hematoxylin/eosin, a mixture of picric acid and fuchsin acid to detect collagen, and after the ZN method, respectively. (D) Multinucleate Langhans giant cells determined in an early non-necrotizing granuloma by ZN staining. (F) Scanty *Mtb* revealed in the caseous matter. Enlarged view of the part of this image with *Mtb* indicated by black arrows is shown in the upper right corner. (G) The tissue specimen of the resected lung (A) was cut into small pieces for producing alveolar macrophages. The diameter of the Petri dish is 10 cm. (H, I) Alveolar macrophages stained by the ZN method after *ex vivo* culture for 18 hours contain *Mtb* in isolation and as a colony, respectively. The black arrows point to alveolar macrophages with acid-fast *Mtb*. The scale bars are 10 μm each.

The absence of a significant number of fibroblasts in the *ex vivo* cultures of cells from all the patients examined suggests that the process of cell isolation did not compromise the integrity of fibrotic tissue containing a large number of necrotizing TB granulomas in the lung specimens used for *ex vivo* expansion of alveolar macrophages (Figs 1B, 4A and 4G, S1A–S1C, S1E–S1G, S1I–S1K and S1M–S1O Fig) or on the histological sections of the lung tissues (Fig 4B–4E). Therefore, neither fibroblasts nor alveolar macrophages can have come from the cells that surround the central part with caseous matter and reside in enclosed necrotizing TB lesions (Fig 4B, 4C and 4E). We propose that alveolar macrophages and other cell types may have come from early, non-fibrotic non-necrotizing TB granulomas, because the *ex vivo* cultures contained multinucleate Langhans giant cells, which are also the markers of early granulomatous inflammatory lesions without the fibrotic capsule and caseous matter in the lungs of TB patients (Fig 4D).

**Fig 5:**
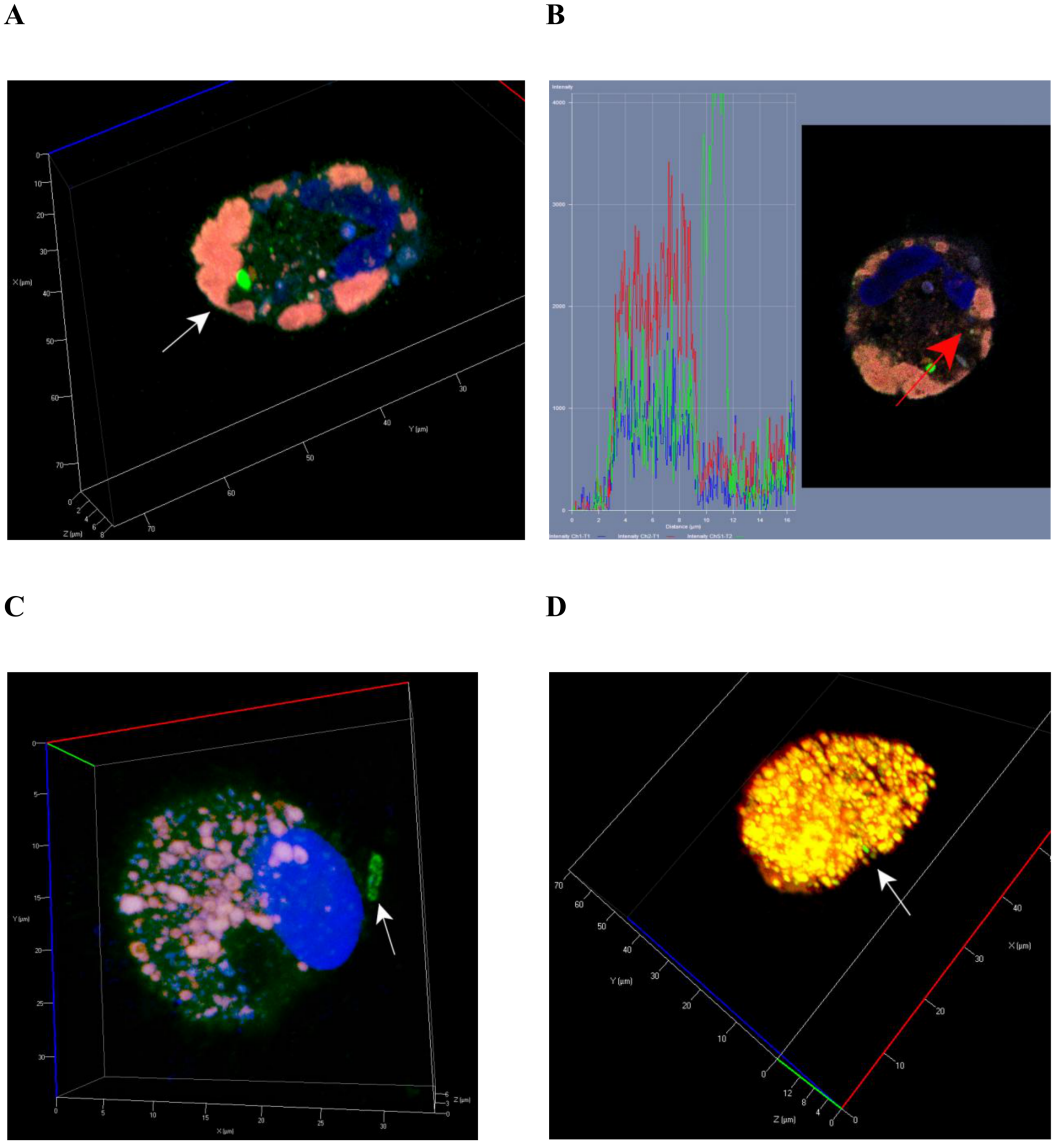
Foamy alveolar macrophages with *Mtb* determined in *ex vivo* cultures. (A, C, D) Representative confocal fluorescent 3D images show lipid-rich foamy alveolar macrophages stained by *Mtb* LAM-specific antibodies (green signal) and Nile red (red signal). Nuclei are stained by DAPI (blue signal). Foamy alveolar macrophages obtained from the resected lungs of patients 4 (A, B), 9 (C), and 15 (C). White arrows point to *Mtb* residing in foamy alveolar macrophages. (B) In the cell profile (A), a foamy alveolar macrophage demonstrates lack of colocalization of *Mtb* and host lipid bodies (lack of yellow signal). However, the markers studied show colocalization for numerous intracellular lipid-laden vesicles (A, B, C, D).

Lipid-rich foamy alveolar macrophages are also important markers of TB inflammation in human lungs [14, 37, 38]. Alveolar macrophages in the *ex vivo* cell cultures were considered as foamy when more than one-third of the cell surface was stained by Nile red. The *ex vivo* cultures of cells obtained from each TB patient had different numbers of Nile red-positive alveolar macrophages, with or without *Mtb* (Fig 5A–5D, S4 Table). The largest lipid-laden vacuoles were detected in foamy alveolar macrophages from the resected lungs of patients 1, 3, 4 (Fig 5A and 5B), and 21.

The *ex vivo* cultures of cells obtained from the resected lungs of all patients were largely composed of alveolar macrophages; however, the number of these cells was varying even though the lung tissue samples were similar in size (Figs 1B and 4G, S1B, S1F, S1J and S1N Fig, S4 Table). Based on histological assessment of the inflammatory response in the lungs to *Mtb* infection coupled with analysis of innate immune response cells, the patients were divided into three groups as in S4 Table. The “high-activation” group included three patients (6, 7, and 10) with multiple morphological signs of the TB process and an unfavorable course of disease at that stage of treatment. The lungs of patients 1, 3, 11, 12, 17, 19, and 21 were found to have morphological signs of a weak TB inflammatory process. Thus these patients were included in the “low-activation” group. The rest of the patients (*n* = 11) were attributed to the “middle-activation” group, because their lungs had morphological signs of TB activity with a risk of progression, should the conditions be unfavorable. The presence of tobacco smokers’ macrophages in the alveolar lumens of the respiratory tissue of the resected lungs had no effect on the form or characteristics of TB lesions, nor had it any on the activation status of TB inflammation in these patients. Note that, in *ex vivo* culture, an increased number of alveolar macrophages (over 200 thousand) was isolated from distant parts of tissue surgically removed from patients both with high/middle (patients 7, 8 and 14) and low (patient 19) activation status, no matter whether they were tobacco smokers or not (patients 7 and 8 were).

Thus, in *ex vivo* culture, CD14-positive alveolar macrophages, foamy or not, as well as with or without tobacco smoke particles, were largely obtained from different (cavity walls or distant) tissue samples from the resected lungs of TB patients with different clinical and X-ray findings, extents of TB disease and activation status of TB inflammation.

### Alveolar macrophages with *Mtb* in the resected lungs of TB patients

By histochemical staining after the ZN method, which detects acid-fast mycobacteria (which by definition have undamaged cell walls), and by immunofluorescent staining with antibodies reacting with the major mycobacterial cell wall component glycolipid lipoarabinomannan (LAM) or with Ag38 (a glycolipoprotein involved in the transport of inorganic phosphate by the ABC transporter of the *Mtb* cell wall and encoded by the *pstS-1* gene [39]), we determined cells with *Mtb* in *ex vivo* cultures of cells obtained from the lung tissue samples of all investigated TB patients. Note that staining human cells after the ZN method allowed *Mtb* to be differentiated immediately both in isolation and in colonies of various morphology. Immunofluorescent staining for *Mtb*, especially those in small colonies, is often required to create confocal 3D imaging of human cells by generating a large number of Z-stacks for an accurate estimate of the number of *Mtb* in these colonies.

**Fig 6:**
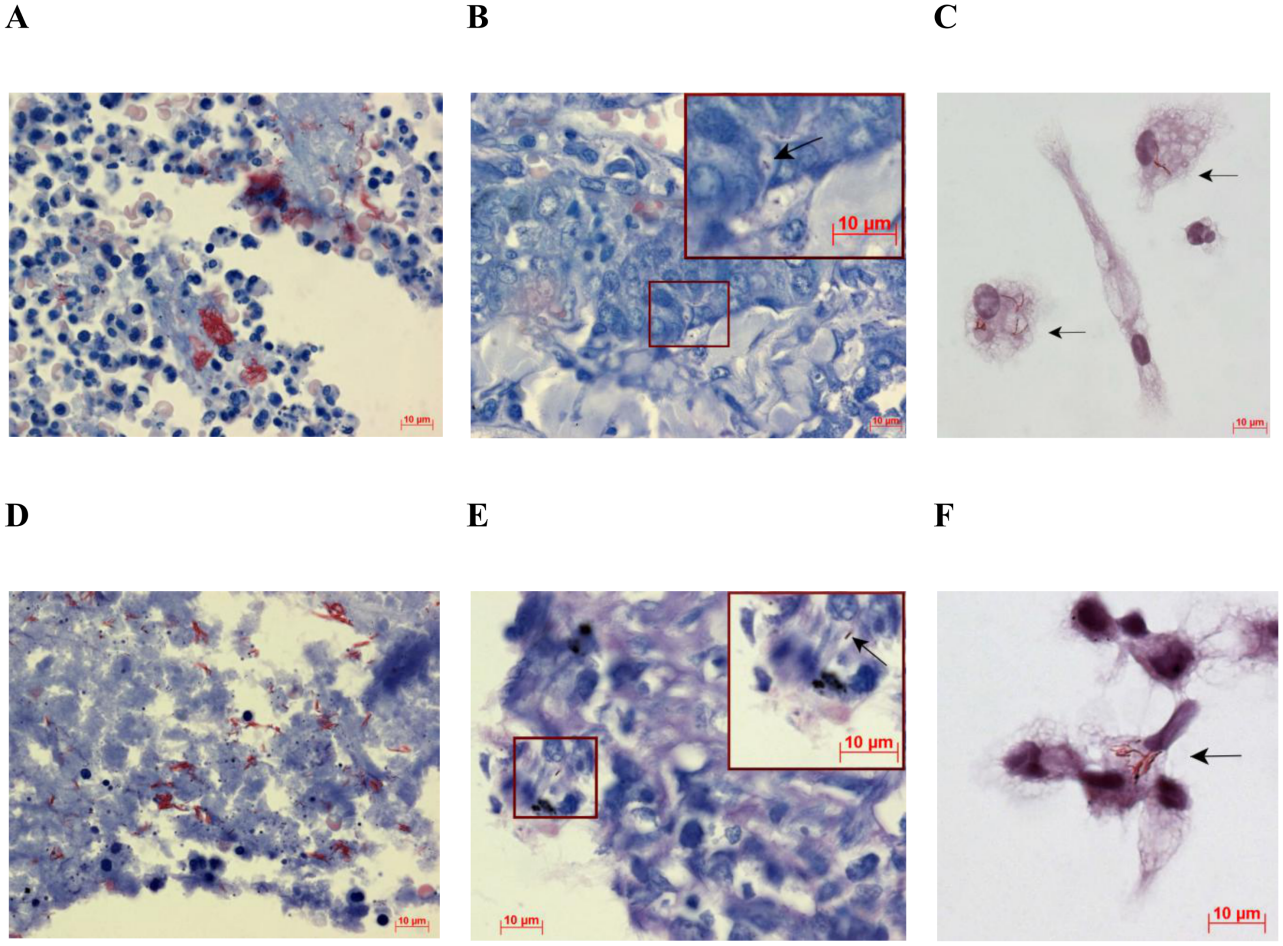
Alveolar macrophages with *Mtb* in colonies are detected both in cavity walls and in the distant parts of lung tissue. (A-C and D-F) Lung tissues and alveolar macrophages obtained from patients 6 and 8, respectively. (A, B, D, E) Representative histological images of the cavity walls (A, D) and the distant parts of lung tissue (B, E) stained by the ZN method demonstrate a massive load of replicating *Mtb* in the cavities and single *Mtb* within alveolar macrophages in other lung regions, respectively. (B, E) Enlarged views of the parts of these images with alveolar macrophages containing single *Mtb* (black arrows) are shown in the upper right corners. (C) Alveolar macrophages obtained from the cavity wall and after *ex vivo* culture for 16 hours contain colonies of replicating *Mtb*. (F) Alveolar macrophages obtained from the distant part of lung tissue and after *ex vivo* culture for 18 hours contain replicating *Mtb* in colonies with cording morphology. Alveolar macrophages (C, F) were stained by the ZN method. The black arrows point to alveolar macrophages with acid-fast *Mtb*. The scale bars are 10 μm each.

*Mtb* were largely found in alveolar macrophages (Figs 1C, 2A–2C, 3A–3C, 4H, 4I and 5A–5D, S1D, S1H, S1L and S1P Fig) and rarely in dendritic cells, neutrophils (Fig 2B), and Langhans giant cells (Fig 2A, S2C Fig). No *Mtb* was found in lymphocytes or fibroblasts. Alveolar macrophages contained individual *Mtb* (Figs 1C, 3A, 4H and 5A–5D, S1P Fig) or *Mtb* in colonies (Figs 2A–2C, 3B, 3C, 4I, 6C and 6F, S1D, S1H and S1L Fig), including those with cording morphology (Figs 2C, 4I, 6C and 6F, S1D Fig), when replicating *Mtb* line up along their longitudinal axes, setting themselves into “braids”. Some *Mtb* colocalized with lipid bodies in foamy alveolar macrophages, some did not (Fig 5A–5C). At the same time, *Mtb* in colonies were detected in alveolar macrophages devoid of lipid bodies. Note that the use of the different staining techniques (ZN and LAM/Ag38 immunofluorescent staining) produced similar numbers of alveolar macrophages with *Mtb* in all cell preparations obtained from the resected lung of each TB patient. Very few individual *Mtb* or *Mtb* in colonies were observed outside human cells in the *ex vivo* cell cultures. Our observation of the morphology of alveolar macrophages and other cells with or without *Mtb* in all *ex vivo* cultures suggests that none of those cells was apoptotic or necrotic (Figs 1C, 2A–2C, 3A–3C, 4H, 4I, 5A–5D, 6C and 6F, S1D, S1H, S1L, S1P and S2A–S2D Figs).

Results of bacteriological and histopathological examination of lung tissue specimens and those of analysis of alveolar macrophages in *ex vivo* cultures are shown, for comparison, in S4 Table. The patients strongly differed in the number of alveolar macrophages with *Mtb* in the *ex vivo* cultures. The largest number of infected alveolar macrophages (up to 38% of the cells examined) was found in the *ex vivo* culture of cells from the cavity wall of patient 6 (Figs 2A–2C and 6C, S1A–S1D Fig). These data are in a good agreement with histological data, which revealed the presence of a large number of infected cells in the cavity wall from this patient’s lung (Fig 6A). Note that Eum and the co-authors [26] reported that the macrophages with *Mtb* from BAL and cavity caseum in Korean TB patients, that is, from lung sites close to cavity walls, made up, on average, about 28% and 24%, respectively. However, most of our TB patients, whose cells were isolated from sites distant from macroscopic lesions, had much fewer infected alveolar macrophages. Their number ranged from 0.11% (patient 12) to 6.54% (patient 9). The presence of mycobacterial IS*6110* insertions was revealed by PCR in the resected lung specimens from all the patients. However, in many patients, it was not so easy to detect infected alveolar macrophages on the histological sections of the distant parts of lung tissue. In patient 8, a large number of cells with *Mtb*, including those in colonies with cording morphology, was observed only in the cavity wall (Fig 6D). The tissues distal to this cavity wall were found to have only few macrophages with individual *Mtb* on the histological sections (Fig 6E). However, the *ex vivo* culture of cells from patient 8 was found to have about 2% of alveolar macrophages infected with *Mtb*, which amounts to about 5 thousand infected cells produced from a relatively small lung tissue specimen (Fig 1B). These alveolar macrophages had either individual *Mtb* (Fig 1C) or *Mtb* in colonies, some with cording morphology (Fig 6F). The histological sections of lung tissue from patient 7 were not found to have cells with *Mtb* (Fig 4D and 4E), while the *ex vivo* culture was found to have about 7 thousand alveolar macrophages with *Mtb*, including those replicating in colonies (Fig 4H and 4I), which amounts to ~3% of all the macrophages obtained from this relatively small lung tissue specimen (Fig 4G). The histological sections of the resected lung tissue from this patient were found to have individual non-replicating acid-fast *Mtb* only in the caseous center of fibrotic necrotizing TB granulomas (Fig 4E and 4F), which is typical of enclosed caseous hypoxic lesions in humans [8, 13, 40]. Note that the smears of tissue homogenates of the resected lungs of patients 7 and 8 were found to have *Mtb* following culture on LJ medium. However, *Mtb* was found to grow on LJ medium only for patient 8, even though the smear of patient 7 contained more mycobacteria than that of patient 8. Similarly, the smear of patient 9 contained more *Mtb* than did that of patient 8. However, *Mtb* failed to grow on LJ medium not only for patient 9, but also for many more others, with or without *Mtb* in the smears of tissue homogenates. In summary, *Mtb* grew on LJ medium only for the lung tissue homogenates of patients 6, 8, 10, 20, with *Mtb* in their smears of tissue homogenates, and patient 11 without *Mtb* in his smear of tissue homogenate. Noteworthy, a very low number of infected alveolar macrophages was observed in the *ex vivo* cultures for patients 11 and 20, while no alveolar macrophages with *Mtb* were found on the histological sections of the distant parts of lung tissue from these and many other patients.

Thus, it can be stated that alveolar macrophages with *Mtb*, including those replicating in colonies, were detected only in *ex vivo* culture, but not by bacteriological or histopathological analyses. With our technique, the number of infected cells in the resected lungs of each patient with pulmonary TB was assessed as rapidly as in two days after surgery.

### Differences in infection parameters between the groups of TB patients

**Fig 7:**
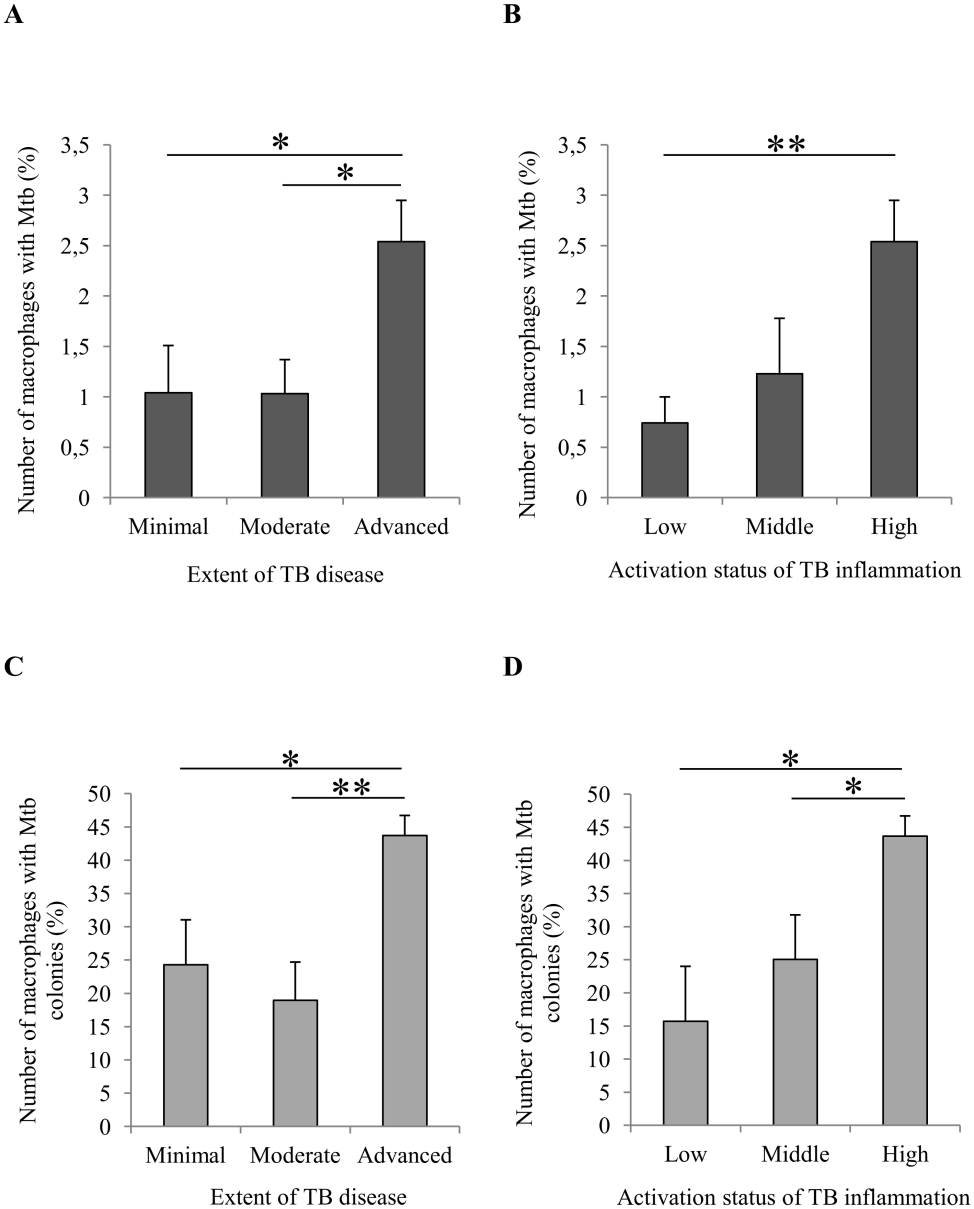
TB patients with the “advanced” extent of disease and “high-activation" status of TB inflammation have a larger population of alveolar macrophages with *Mtb* and mycobacterial colonies in the distant parts of lung tissue. (A, B) The number of alveolar macrophages with *Mtb* (in isolation or as colonies) expressed as the percentage of the total number of alveolar macrophages analyzed. (C, D) The number of alveolar macrophages with *Mtb* in colonies expressed as the percentage of the total number of alveolar macrophages with any *Mtb*. Data are expressed as the means ± SEM. *P < 0.05; **P < 0.01, Student’s *t*-test.

**Fig 8:**
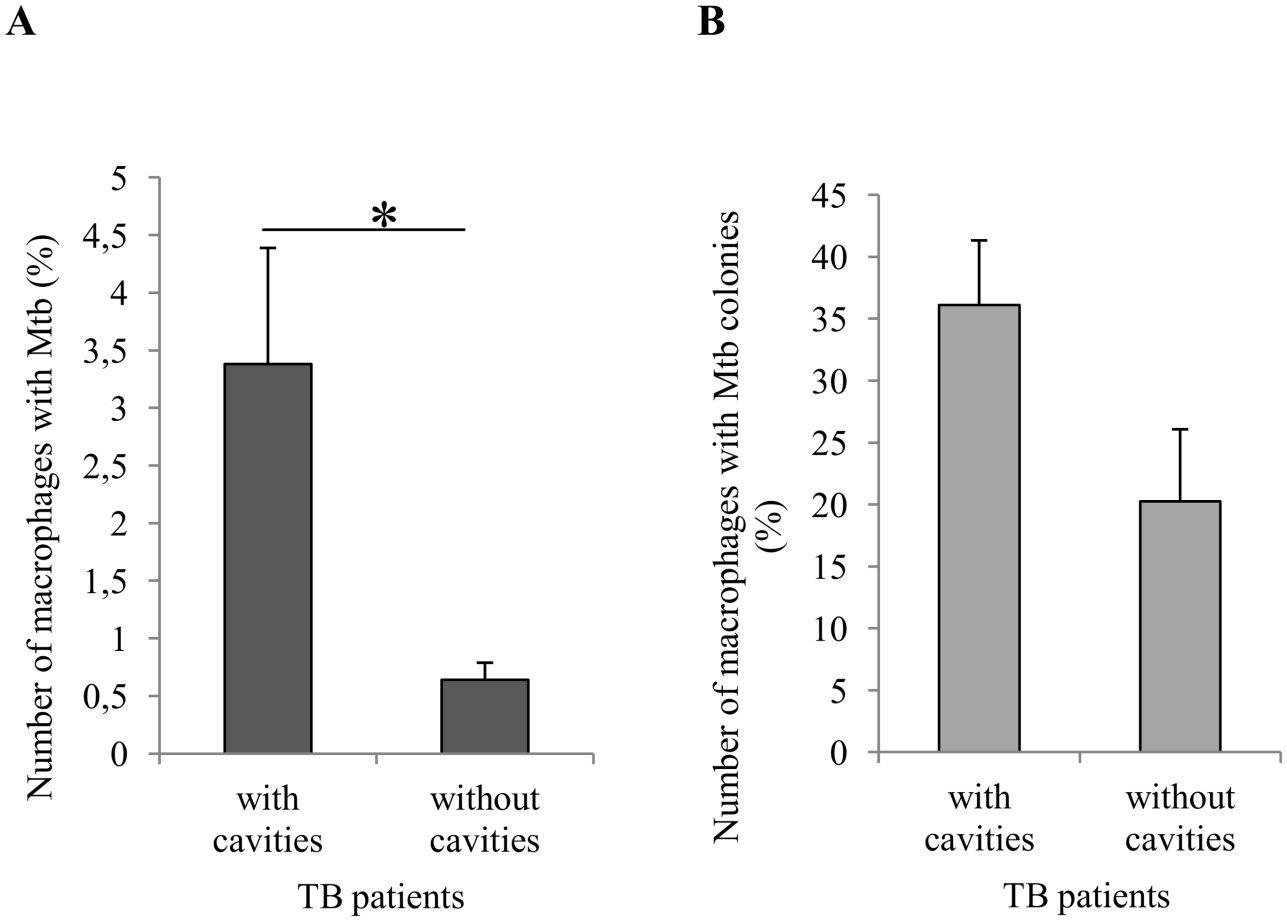
A significant increase in the number of alveolar macrophages with *Mtb* in the distant parts of lung tissue in patients with cavities. (A) The number of alveolar macrophages with *Mtb* (in isolation or as colonies) expressed as the percentage of the total number of alveolar macrophages analyzed. (B) The number of alveolar macrophages with *Mtb* in colonies expressed as the percentage of the total number of alveolar macrophages with any *Mtb*. Data are expressed as the means ± SEM. *P < 0.05, Student’s *t*-test.

It was found that, after the surgery of patients with pulmonary TB, the number of alveolar macrophages containing either whatever *Mtb* (individual or as colonies) or replicating *Mtb* in colonies had changed in the *ex vivo* cultures of lung tissue from distant parts (S4 Table). A comparison of infection parameters in TB patients with different extents of TB disease as in S1 Table and different activation status of TB inflammation as in S3 Table demonstrated that a considerable increase in the number of alveolar macrophages with *Mtb* (individual or as colonies) and with replicating microbes occurred only in patients 7 and 10 (“advanced” and “high-activation” groups), who had cavities (Fig 7A–7D). No statistically significant changes in these parameters were found between the “minimal” (*n* = 13) and “moderate” (*n* = 5) groups of patients or between the “low-activation” (*n* = 7) and “middle-activation” (*n* = 11) groups of patients (Fig 7A–7D). A substantially higher number of infected alveolar macrophages was in the patients with than without cavities, *n* = 4 and *n* = 16, respectively (Fig 8A). Just to remind, patients 8 and 9, who had cavities, were "moderate” and “minimal”, respectively, and “middle-activation” both. The difference in the number of alveolar macrophages with replicating *Mtb* between these groups of patients was not statistically significant (Fig 8B). Smokers with pulmonary TB had a higher number of alveolar macrophages with *Mtb* as compared with nonsmoking TB patients, *n* = 17 and *n* = 3, respectively (S3A Fig). Note that all the patients with cavities were tobacco smokers. At the same time, the *ex vivo* culture of cells from the resected lung of patient 13 with chronic obstructive pulmonary disease was found to contain only few infected cells from among more than 100 thousand smokers’ macrophages examined in it (S3 Table). No statistically significant difference in the number of alveolar macrophages with replicating *Mtb* was found between smokers and nonsmokers (S3B Fig). Also, there were no statistically significant differences in the number of foamy alveolar macrophages between all the groups of TB patients (S4A–S4D Fig), including the groups with a different number of *Mtb*-infected alveolar macrophages in the resected lungs, namely, less or more than 1% out the total number of macrophages examined, *n* = 13 and *n* = 7, respectively (S4E Fig).

Thus, the highest number of infected alveolar macrophages in *ex vivo* culture was in the cavity wall and in the tissue samples obtained from the distant parts of the surgically removed lung tissue of TB patients with cavities, no matter how different the infection process in these patients was.

## Discussion

*M*. *tuberculosis* is spread by airborne transmission and largely invades alveolar macrophages in the lungs [2–6]. The capacity of *Mtb* to evade the immune response of the host organism and drug treatment makes this bacterium a very successful and widespread human pathogen causing an infectious disease, which is so difficult to cure [1–3]. Today, as TB remains to be a leading global health issue, new technologies are required for analysis of relationships between the pathogen and host cells in TB patients’ lungs for assessing the efficiency of anti-TB chemotherapy, for considering a personalized revision of treatment regimens and for making predictions as to how the tuberculosis infection will proceed in each TB patient [28].

Here, we propose a technique to develop *ex vivo* cultures of cells from different parts of lung tissues surgically removed from patients with pulmonary TB. This technique allowed us to determine rapidly (in two days after surgery) the level of infection with *Mtb* in the cells of the resected lungs and, by the presence or absence of *Mtb* colonies, the functional status of the TB agent at the time of surgery. The *ex vivo* cultures of cells from cavity wall and distant parts of the resected lungs mostly contained alveolar macrophages, while those of cells from the BAL fluid, sputum and cavity caseum in Korean TB patients, neutrophils [26]. The observed differences in cell composition between these cultures might be both due to some individual features of TB patients and probably because neutrophils are easier to wash away from the lung tissues with the methods the authors used. As a result, although neutrophils and dendritic cells were also present in the *ex vivo* cell cultures, the main host cells for *Mtb* in the studied TB patients were CD14-positive alveolar macrophages. Our data are consistent with the opinion [2–6] that the main modulators of TB infection in affected humans are alveolar macrophages, which are long-living cells with a minimum half-life of 4 months [41], rather than the broadly discussed neutrophils [34, 35, 42] or dendritic cells [43, 44].

Interestingly, we did not observe phagosomes with engulfed lymphocytes, erythrocytes, or platelets in alveolar macrophages or other cells in the *ex vivo* cell cultures. The result is surprising, because the pellets obtained by centrifugation of the lung homogenates and used for *ex vivo* expansion of alveolar macrophages contained a large number of blood cells too. In our studies of granulomas of mice with latent TB infection we detected many phagosomes with engulfed lymphocytes and platelets at various stages of degradation in mouse granuloma macrophages in the *ex vivo* culture [29, 30]. Therefore, it is necessary to proceed with studying capabilities for phagocytosis in alveolar macrophages obtained from the resected lung tissues of TB patients.

We have found that the number of infected alveolar macrophages strongly varied across TB patients. Interestingly, an increased number of *Mtb*-infected alveolar macrophages in the distant parts of the resected lungs correlated, first of all, with the presence of cavities, the walls of which were observed to have a substantial *Mtb* load both in *ex vivo* cell cultures and on histological sections. It can therefore be hypothesized that *Mtb* residing in the cavity walls, where their active replication is promoted by increased access to oxygen, can not only be disseminated outside the organism with sputa coughed out [7, 8, 13, 15], but also reach the lung parts distant from cavities and, probably, other organs, migrating with alveolar macrophages. As was determined using the zebrafish model [45, 46], macrophages can migrate—actively and in a targeted manner—which makes gather at the foci of TB infection and inflammation. Our studies of mouse granuloma macrophages with BCG mycobacteria using an *ex vivo* model demonstrated that the migratory activity of these cells does not depend on the number of mycobacteria in them [11, 29]. The capacity of alveolar macrophages with *Mtb* to migrate from cavity walls to distant parts is in part supported by the presence of colonies of *Mtb* with cording morphology, similar to colonies in the cavity walls in the same patients. Overall, the travel of *Mtb*-infected cells across TB patients’ lungs requires further research, probably using safe fluorescent labels delivered, say, with aerosols to the cavities before surgery and then testing alveolar macrophages from different parts of the resected lungs for these labels. On the whole, the presence of a large number of neither apoptotic nor necrotic alveolar macrophages with *Mtb* in the *ex vivo* cultures of cells from the lungs of TB patients with cavities, no matter to what extent the host cells are infected by *Mtb*, suggests that these patients require special attention. The use of anti-TB drugs or host-directed therapies that aim to eliminate *Mtb* in alveolar macrophages residing in cavity walls should prevent *Mtb* dissemination outside and throughout TB patients’ organisms and thus reduce the risk of extrapulmonary TB.

During isolation of cells from the resected lungs of TB patients, we discarded fibrotic TB foci with caseous matter inside, these foci being the main hallmark of TB inflammation in human lungs after *Mtb* infection [8, 13]. The absence of extracellular *Mtb* and fibroblasts in the *ex vivo* cultures suggests that, however damaging to cells the isolation process may be, the integrity of these TB lesions was not compromised. Thus we failed to obtain extracellular *Mtb* or alveolar macrophages with *Mtb* from these fibrotic TB lesions in *ex vivo* culture. Therefore, fibrotic granulomatous TB lesions are not only reservoirs for *Mtb* residing within the caseum of necrotic central foci [13, 47], but they also protect the lungs from further *Mtb* dissemination at a particular stage of infection [4, 48]. Our observation is also supported by a relatively low number of *Mtb*-infected cells in the *ex vivo* culture of cells from the resected lungs of most TB patients without cavities. Therefore, the treatment of TB patients as these should be aimed at preventing at preventing encapsulated hypoxic TB lesions with scanty *Mtb* from evolving into open cavitary lesions heavily loaded with replicating *Mtb*.

Another hallmark of *Mtb* infection in the lungs of TB patients is lipid-laden foamy alveolar macrophages, which are thought to promote pathogen survival and replication [14, 37, 38]. However, in our study, the number of foamy alveolar macrophages in the resected lungs strongly varied across TB patients and did not correlate with the particular TB disease characteristics or with the level of infection by *Mtb* in alveolar macrophages in *ex vivo* culture. Note that the main conclusions about the effect of lipid-rich macrophages on *Mtb* infection progression were previously made only by experimenting with *in vitro* infection of cells with *Mtb* strain H37Rv and by *in vivo* studies with animal models [37, 49]. As has been demonstrated (for our contribution, see [30]), *in vitro* infection of mouse cells and *in vivo* infection of animals lead to different, even opposing, responses of mycobacteria and macrophages. Also, the hypothesis that foamy alveolar macrophages are directly responsible for the development of cavities in the lungs of TB patients was solely based on the results of histological analysis of some human autopsy specimens and did not consider levels of infection with *Mtb* in the cells of these lung tissues [50, 51]. Therefore, it is necessary to collect more data about relationships between foamy alveolar macrophages and *Mtb* in the lungs of TB patients, because adjunctive host-directed therapy proposed for treatment of TB includes the use of statins, which inhibit the biosynthesis of cholesterol in host cells [52, 53].

Thus we found alveolar macrophages with *Mtb* in the *ex vivo* cultures of cells from the resected lungs of even those TB patients, whose sputum smears and lung tissue homogenates did not contain acid-fast mycobacteria or reveal growing *Mtb* colonies on LJ medium. The fact that the lung tissue specimens, which were similar in size, yielded different numbers of alveolar macrophages in *ex vivo* culture could probably serve as a marker of an inflammatory response to *Mtb* infection in the lungs of TB patients. However, to confirm this, further research is required. On the whole, we detected a small number of *Mtb*-infected alveolar macrophages isolated from sites distant from macroscopic lesions in the resected lungs of TB patients. Our data are consistent with the hypothesis that explains the absence of alveolar macrophages with *Mtb* on the histological sections without cavities by their low number in the lung tissue [54]. The detection of alveolar macrophages with acid-fast *Mtb* in *ex vivo* cultures as soon as 16–18 h after isolation of cells from the resected lungs of all TB patients suggests that the technique proposed for assessing the level of infection in alveolar macrophages in the resected lungs of TB patients has higher sensitivity than do bacteriological or pathomorphological methods. The use of our technique demonstrates that *Mtb* in the alveolar macrophages of TB patients’ lungs remain to be acid-fast, although it was previously believed—because these *Mtb* were not detected by standard analyses—that, in human lungs, the mycobacterial cell walls undergo substantial changes and *Mtb* lose acid-fastness [8, 54].

In summary, the observation of a large number of alveolar macrophages with *Mtb*, including those in colonies, in *ex vivo* cultures clearly indicates that the efficiency of the anti-TB therapy given some patients (in particular, patients 3 and 6÷10) before surgery was low and that the treatment regimens these patients receive during the post-surgery period should be heavily revised, no matter which TB disease level they have. A small number of alveolar macrophages with non-replicating *Mtb* detected in the *ex vivo* cultures of cells from patients 1, 2, 11, 12, 15, 20, and 21 indicates that the anti-TB therapy given them was chosen correctly and applied accurately, and so good outcome should be expected. As far as the other patients, who had been given a relatively efficient anti-TB therapy before surgery and thus had not developed cavities, are concerned, they appear—based on the number of *Mtb*-infected alveolar macrophages in the *ex vivo* cultures—to be still in need of close monitoring and better treatment regimens to eliminate replicating *Mtb*. Thus, the number of *Mtb*-infected alveolar macrophages in the *ex vivo* cultures of cells obtained from resected lungs can be used, probably, as a new biomarker of predictive value for assessing treatment efficiency in relation to TB patients before surgery. Furthermore, the *ex vivo* cell cultures developed are suitable for quick estimation of *Mtb* virulence (see [31]) and exploring individual points of relationships between alveolar macrophages and *Mtb* in the lungs of TB patients with different characteristics of TB disease. Alveolar macrophages containing *Mtb* of different functional status and obtained in large numbers from *ex vivo* cultures of cells from some TB patients can be used for individualized testing of drugs, both existing and emerging, within the concepts of personalized medicine and adjunctive host-directed therapies [18, 28, 55, 56]. The appropriate choice of treatment regimen, which takes into account the biological properties and functional status of a pathogen in alveolar macrophages in a separate TB patient’s lungs, is believed to prevent exacerbation [2, 8].

## Conclusions

The reported technique for developing *ex vivo* cultures of alveolar macrophages allows the level of infection with *Mtb* in the host cells in the lungs of post-operative patients with pulmonary TB to be assessed and the functional status of a pathogen to be determined in a matter of unprecedentedly short time. In summary, the proposed method of analysis can be used as the basis for the development of individual strategies in post-operative case treatment and making decisions as to how further prophylactic, epidemiological and medical treatment measures against pulmonary TB should be applied to patients and, if needed, revised.

## Supporting information

S1 Fig Cell suspension containing alveolar macrophages is isolated from granulomatous fibrotic lung tissue. (A, E, F, M) Lung parts surgically removed from patients 6, 14, 19, and 20, respectively. (B, F, J, N) Tissue specimens with cavity wall (B) and distant parts (F, J, N) from the resected lungs (A, E, F, M), respectively. Petri dishes are each 10 cm in diameter. (C, G, K, O) Cell suspensions containing alveolar macrophages were obtained from the lung specimens (B, F, J, N), respectively, and separated from closed caseous tuberculous lesions in the fibrous capsule staying in the sieves. (D, H, L, P) Alveolar macrophages obtained from cell suspensions (C, G, K, O), respectively, and stained by the ZN method after *ex vivo* culture for 16–18 hours. The black arrows point to alveolar macrophages with acid-fast *Mtb*. The scale bars are 10 μm each.
(TIFF)

S2 Fig Multinucleate Langhans giant cells are determined in *ex vivo* cultures of alveolar macrophages. (A, B, C, D) Langhans giant cells obtained from the lung tissue of patients 2, 7, 8, and 11, respectively, and stained by the ZN method. The black arrow points to a Langhans giant cell with acid-fast *Mtb*. The scale bars are 10 μm each.
(TIFF)

S3 Fig A significant increase in the number of alveolar macrophages with scanty *Mtb* is determined in the distant parts of lung tissue in smoking TB patients. (A) The number of alveolar macrophages with *Mtb* (in isolation or as colonies) expressed as the percentage of the total number of alveolar macrophages analyzed. (B) The number of alveolar macrophages with *Mtb* in colonies expressed as the percentage of the total number of alveolar macrophages with any *Mtb*. Data are expressed as the means ± SEM. **P < 0.01, Student’s *t*-test.
(TIFF)

S4 Fig No differences between the patient groups in the mean population size of foamy alveolar macrophages (Mph) in the distant parts of lung tissue are found. (A, B, C, D, E) The number of foamy alveolar macrophages expressed as the percentage of the total number of alveolar macrophages analyzed. Data are expressed as the means ± SEM. (E) Data on patients differing in the number of alveolar macrophages with *Mtb* obtained from resected lungs and presented as the percentage of the total number of macrophages examined.
(TIFF)

S1 Table The characteristics of the patients with pulmonary TB before surgery. (PDF)

S2 Table The extents of TB disease for the patients before surgery. (PDF)

S3 Table Cell populations obtained *ex vivo* from the resected lungs of TB patients. (PDF)

S4 Table Comparison of the different methods used for analysis of alveolar macrophages (Mph) with *M*. *tuberculosis* (*Mtb*) in the resected lungs of patients with pulmonary TB. (PDF)
