# Supplementary figures and images for: Ex vivo expansion of alveolar macrophages with Mycobacterium tuberculosis from the resected lungs of patients with pulmonary tuberculosis
Source: PLoS One. 2018 Feb 5;13(2):e0191918. doi: 10.1371/journal.pone.0191918 (PMC5798839; doi:10.1371/journal.pone.0191918)

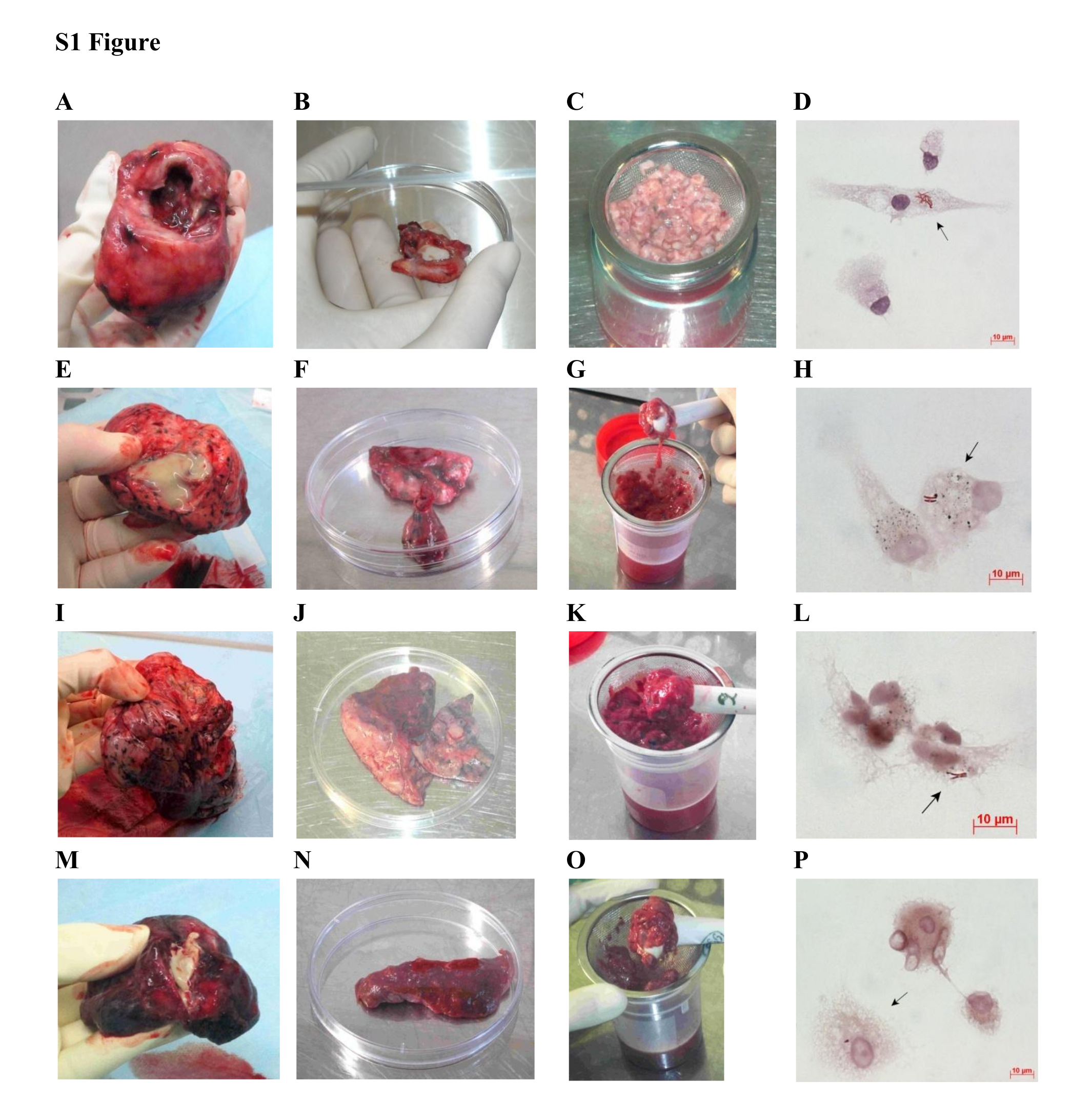

Supplement: S1 Fig — (A, E, F, M) Lung parts surgically removed from patients 6, 14, 19, and 20, respectively. (B, F, J, N) Tissue specimens with cavity wall (B) and distant parts (F, J, N) from the resected lungs (A, E, F, M), respectively. Petri dishes are each 10 cm in diameter. (C, G, K, O) Cell suspensions containing alveolar macrophages were obtained from the lung specimens (B, F, J, N), respectively, and separated from closed caseous tuberculous lesions in the fibrous capsule staying in the sieves. (D, H, L, P) Alveolar macrophages obtained from cell suspensions (C, G, K, O), respectively, and stained by the ZN method after ex vivo culture for 16–18 hours. The black arrows point to alveolar macrophages with acid-fast Mtb. The scale bars are 10 μm each. (TIFF) [file pone.0191918.s001.tiff]

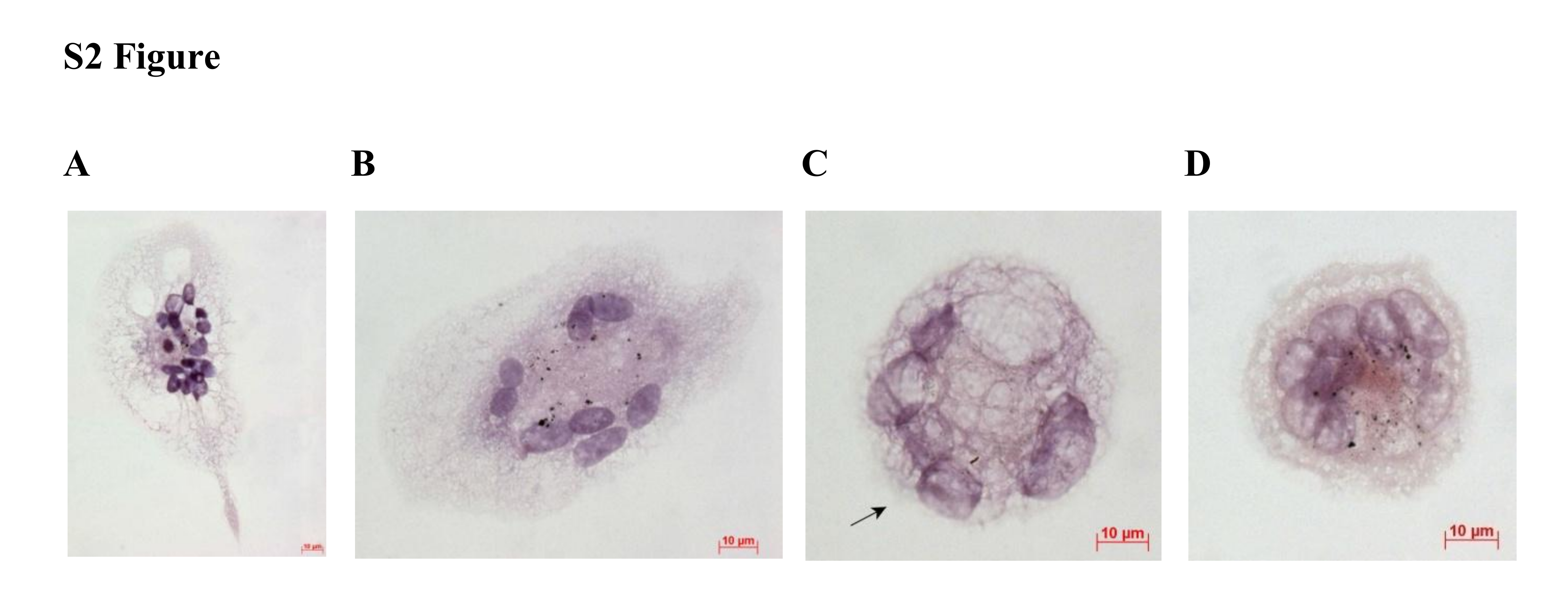

Supplement: S2 Fig — (A, B, C, D) Langhans giant cells obtained from the lung tissue of patients 2, 7, 8, and 11, respectively, and stained by the ZN method. The black arrow points to a Langhans giant cell with acid-fast Mtb. The scale bars are 10 μm each. (TIFF) [file pone.0191918.s002.tiff]

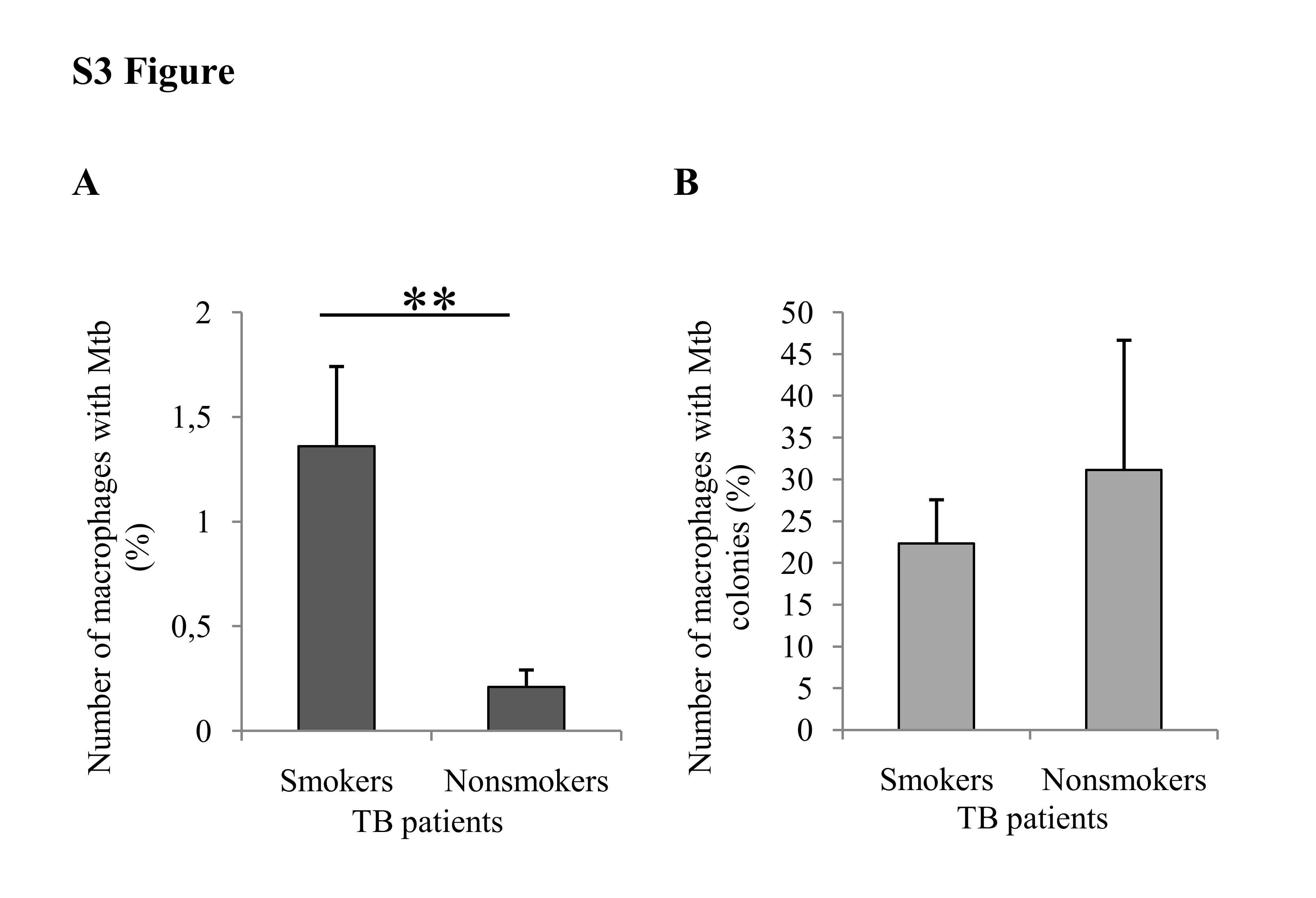

Supplement: S3 Fig — (A) The number of alveolar macrophages with Mtb (in isolation or as colonies) expressed as the percentage of the total number of alveolar macrophages analyzed. (B) The number of alveolar macrophages with Mtb in colonies expressed as the percentage of the total number of alveolar macrophages with any Mtb. Data are expressed as the means ± SEM. **P < 0.01, Student’s t-test. (TIFF) [file pone.0191918.s003.tiff]

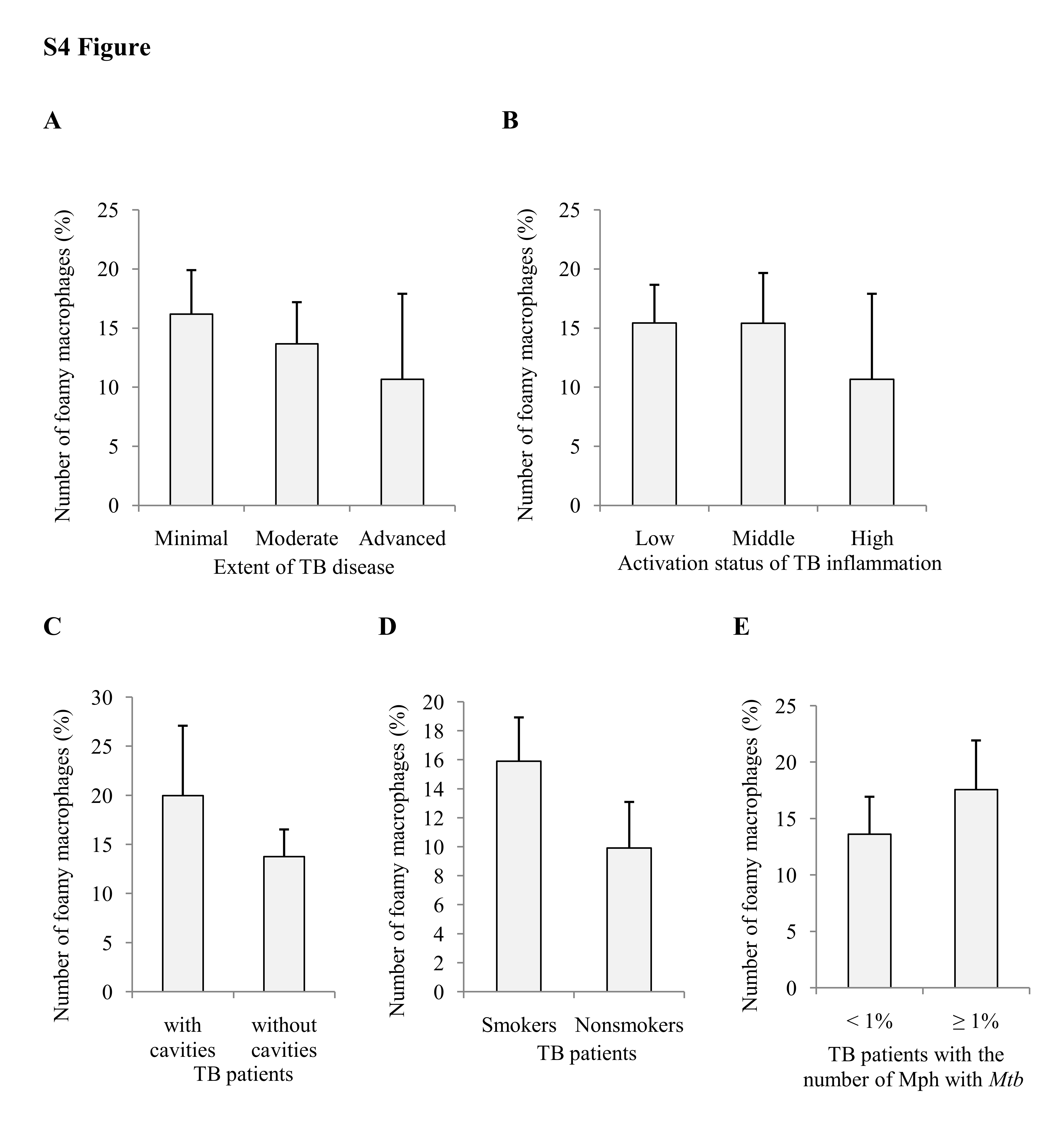

Supplement: S4 Fig — (A, B, C, D, E) The number of foamy alveolar macrophages expressed as the percentage of the total number of alveolar macrophages analyzed. Data are expressed as the means ± SEM. (E) Data on patients differing in the number of alveolar macrophages with Mtb obtained from resected lungs and presented as the percentage of the total number of macrophages examined. (TIFF) [file pone.0191918.s004.tiff]
