# Supplementary material for: Ex vivo expansion of alveolar macrophages with Mycobacterium tuberculosis from the resected lungs of patients with pulmonary tuberculosis
Source: PLoS One. 2018 Feb 5;13(2):e0191918. doi: 10.1371/journal.pone.0191918 (PMC5798839; doi:10.1371/journal.pone.0191918)
Supplement: S1 Table — (PDF) [file pone.0191918.s005.pdf]

S1 Table. The characteristics of the patients with pulmonary TB before surgery.

Patient no.	Age (ye)	Sex[c]	Treatment before surgery		Smear of sputum[f]	The character of TB lesions[g]			TB disease level[h]	Surgery[i]	HIV infec-tion[j]	Attendant disease[k]
			Drugs[d]	Mo[e]		Caseation	Cavitation	Fibrosis				
1[a]	27	M	Z Pt Cap Pas Cs Of	24	-	+	-	+	Min	S6 LL	-	
2	59	M	H R Z Pt Cap	9	-	+	-	+	Min	UL LL	-	
3[a]	36	M	H Z Pt Cap Pas Cs	9	-	+	-	+	Min	UL RL	-	
4	33	M	H R Z E	5	-	+	-	+	Min	S1-2 RL	-	Hepatitis C
5	20	M	H R Z E Pt Cap Cs	11	-	+	-	+	Mod	S1-3 LL	-	
6[b]	38	M	Z E Pt Cap Cs	155	+	+	+	+	Adv	S2,6 RL	-	
7[a]	32	M	H Z E Pt Cap Cs	54	+	+	+	+	Adv	UL RL	+	Hepatitis C
8[a]	34	M	H Z E Pt Rfd	5	+	+	+	+	Mod	S1-3 LL	-	Diabetes
9[a]	23	M	Z Cap Pas Cs Rfd	31	-	+	+	+	Min	S1-3 LL	-	Diabetes
10[a]	45	F	Z E Pt Cap Pas Cs	41	+	+	+	+	Adv	RL	-	

11[a]	43	M	Z, Pt, Cap, Pas, Cs	74	-	+	-	+	Min	S1-2 RL	-	
12[a]	51	F	Z Pt Cap Pas Cs	19	-	+	-	+	Min	UL RL	-	
13	59	M	H R Z Pt Pas	3	-	+	-	+	Min	S2 LL	-	COPD
14[a]	38	M	R Z E Pas	6	-	+	-	+	Min	S6 LL	-	
15[a]	25	M	H R Z E	58	-	+	-	+	Mod	S1-3 LL	-	
16[a]	43	M	Z Pt Cap Pas Cs Of	40	-	+	-	+	Mod	UL RL	-	
17[a]	24	F	H Z E Rfd	5	-	+	-	+	Min	S1-2 LL	-	
18[a]	28	M	H R Z E	27	-	+	-	+	Min	S1-2 LL	-	
19[a]	36	M	Z Pt Cap Pas Cs	26	-	+	-	+	Min	S1-2 RL	-	
20[a]	33	F	H Z E Rfd	113	-	+	-	+	Mod	S6 RL	+	Hepatitis C
21[a]	33	F	Z E Pas Cs Of	14	-	+	-	+	Min	S6 RL	-	

[a]MDR TB.

[b]XDR TB.

[c]M, male; F, female.

[d]H, isoniazid; R, rifampicin; Z, pyrazinamide; E, ethambutol; Pt, protionamide; Cap, capreomycin; Pas, para-aminosalicylic acid; Cs, cycloserine; Of, ofloxacin; Rfd, rifabutin.

[e]Duration of therapy.

[f]Acid-fast mycobacteria detected at the time of surgery. (+), *Mtb* positive; (-), *Mtb* negative.

[g]Chest radiography; (+), yes; (-), no.

[h]Different extents of TB disease based on clinical manifestations and chest radiography: Min, “minimal”; Mod, ”moderate”; Adv, “advanced” (explanation in S2 Table).

[i]S, segment; UL, upper lobe; LL, left lung; RL, right lung.

[j](+), yes; (-), no.

[k]COPD, chronic obstructive pulmonary disease.
