# Supplementary material for: Ex vivo expansion of alveolar macrophages with Mycobacterium tuberculosis from the resected lungs of patients with pulmonary tuberculosis
Source: PLoS One. 2018 Feb 5;13(2):e0191918. doi: 10.1371/journal.pone.0191918 (PMC5798839; doi:10.1371/journal.pone.0191918)
Supplement: S2 Table — (PDF) [file pone.0191918.s006.pdf]

S2 Table. The extents of TB disease for the patients before surgery.

TB disease	"advanced"	"moderate"	"minimal"
Computed Tomography	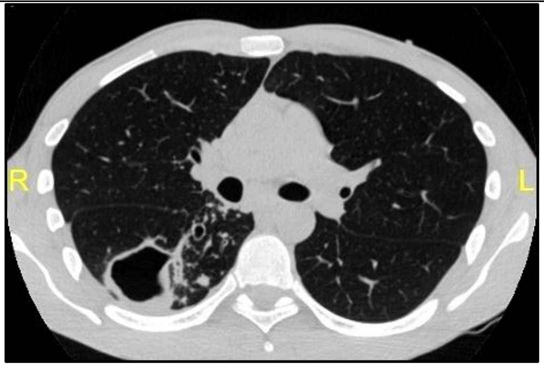 (patient 10)	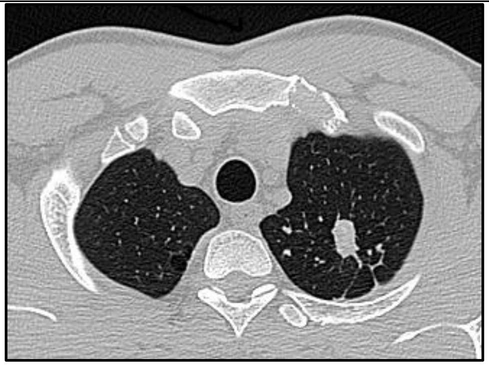 (patient 8)	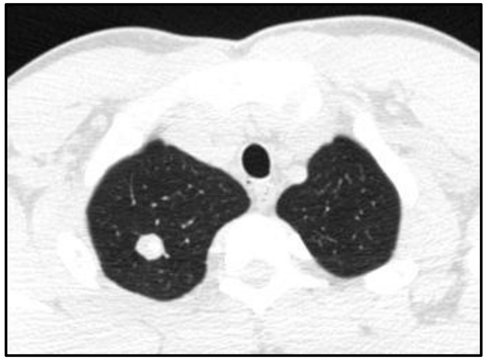 (patient 11)
TB lungs[a]	+++	++	+
TB symptoms[b]	++	+	-
Sputum[c]	+	+/-	-

[a]TB lung tissue destruction detected by chest radiography and Computed Tomography: (+++) extended in many lung segments; (++) limited to the terminal part of lung segments; (+) solitary in one lung segment.

[b]Clinical sings of TB disease are expressed: (++) well; (+) slightly; (-) not markedly.

[c]*M. tuberculosis* in sputum: (+) yes; (-) no.
