# Supplementary material for: Ex vivo expansion of alveolar macrophages with Mycobacterium tuberculosis from the resected lungs of patients with pulmonary tuberculosis
Source: PLoS One. 2018 Feb 5;13(2):e0191918. doi: 10.1371/journal.pone.0191918 (PMC5798839; doi:10.1371/journal.pone.0191918)
Supplement: S3 Table — (PDF) [file pone.0191918.s007.pdf]

S3 Table. Cell populations obtained *ex vivo* from the resected lungs of TB patients.

Patient no.	Macrophages[a]	Dendritic cells[a]	Langhans giant cells[a]	Fibroblasts[a]	Neutrophils[a]	Lymphocytes[a]
1	99.47*	-	0.53	-	-	-
2	97.7*	-	0.68	1.22	-	0.41
3	97.97*	0.95	1.02	-	0.07	-
4	99.11*	0.55	0.22	0.06	0.06	-
5	96.25*	2.62	-	-	-	1.12
6	93.94*	2.27	0.43	-	0.5	2.87
7	94.45*	4.91	0.25	0.03	0.05	0.3
8	87.57*	8.7	0.04	-	0.97	2.73
9	97.77*	1.11	0.3	-	0.41	0.41
10	73.44*	1.05	0.01	1.64	22.46	1.4
11	97.35*	-	2.72	-	-	-

12	94.55	4.87	-	-	-	0.61
13	90.33*	7.43	0.01	-	-	2.23
14	96.23*	3.4	0.06	0.04	0.08	0.19
15	96.4*	1.78	0.07	0.06	1.25	0.44
16	94.97*	2.23	0.01	0.22	1.9	0.66
17	99.87*	-	0.13	-	-	-
18	91.48	8.36	0.01	0.07	0.07	-
19	98.71	1.28	0.01	-	-	-
20	99.13*	0.73	0.01	0.11	-	0.03
21	98.34*	-	0.01	-	1.65	-

[a]Data are presented as the percentage of the number of cells of a particular type out of the total number of cells examined.

*Smokers' alveolar macrophages.
