# Supplementary material for: Ex vivo expansion of alveolar macrophages with Mycobacterium tuberculosis from the resected lungs of patients with pulmonary tuberculosis
Source: PLoS One. 2018 Feb 5;13(2):e0191918. doi: 10.1371/journal.pone.0191918 (PMC5798839; doi:10.1371/journal.pone.0191918)
Supplement: S4 Table — (PDF) [file pone.0191918.s008.pdf]

S4 Table. **Comparison of the different methods used for analysis of alveolar macrophages (Mph) with *M. tuberculosis* (*Mtb*) in the resected lungs of patients with pulmonary TB.**

Patient no.	Bacteriology (2 months)[a]			Histopathologic examination (3 months)[a]				Cells in *ex vivo* culture (2 days)[a]					
	Mtb in smear of tissue homogenate[b]	PCR-analysis[c]	*Mtb* colonies on LJ medium[d]	Alveolar Mph with *Mtb*[e] in			TB inflammation[h]	Lung tissue for processing of alveolar Mph[i]	Mph[j], *n*	Alveolar Mph with			Foamy alveolar Mph[n], %
				Early granulomas[f]	Caseous granulomas[g]	Wall of cavities				*Mtb*[k], %	*Mtb*[l], *n*	*Mtb* in colonies[m], %	
1	-	+	-	-	-		L	Distant	13536	0.53	62	0	21.74
2	-	+	-	-	-		M	Distant	17304	0.42	78	0	6.45
3	+	+	-	-	-		L	Distant	32424	2.07	664	25.0	22.58
4	+	+	-	-	-		M	Distant	42960	0.56	246	60.0	42.86
5	+	+	-	-	-		M	Distant	9252	1.95	122	20.0	9.09
6	+++	+	+++	+	+	+	H	Cavity wall	63624	37.72	24267	72.0	28.11

7	+++	+	-	-	-	+	H	Distant	224760	3.04	6791	47.37	1.82
8	+	+	+++	+	+	+	M	Distant	269592	1.88	5064	36.49	19.08
9	++	+	-	+	+	+	M	Distant	23160	6.54	1502	20.63	39.47
10	++	+	+++	-	-	+	H	Distant	22608	2.4	533	40.0	19.51
11	-	+	+	-	-		L	Distant	5292	0.68	39	0	1.64
12	-	+	-	-	-		L	Distant	16707	0.11	16	0	7.69
13	+	+	-	-	-		M	Distant	110880	0.13	84	0	6.33
14	+	+	-	-	-		M	Distant	237960	0.36	817	66.67	2.11
15	+	+	-	-	-		M	Distant	30600	0.47	116	25.0	23.91
16	+	+	-	-	-		M	Distant	85104	0.63	526	13.33	2.56
17	+	+	-	-	-		L	Distant	44892	1.28	537	25.0	11.4
18	+	+	-	-	-		M	Distant	58320	0.37	205	33.33	5.14
19	+++	+	-	-	-		L	Distant	371412	0.15	321	60.0	16.89

20	+	+	+	-	-		M	Distant	32664	0.22	89	0	13.68
21	+	+	-	-	-		L	Distant	6432	0.37	21	0	26.09

[a]Time necessary for analysis.

[b, e, k, m]Acid-fast mycobacteria detecting.

[b](+++), numerous; (++), moderate; (+), small; (-), none.

[c](+), IS*6110* probe positive.

[d]LJ, Lowenstein-Jensen. *Mtb* growth: (+++), numerous; (+), scanty; (-), none.

[f]The non-necrotizing granulomas without peripheral fibrosis. (+), rarely identified cells.

[g]The classic closed TB lesions with caseation necrosis in center surrounded by a rim of the macrophages and finally by peripheral fibrosis. (+), rarely identified cells.

[h]Activation status of TB inflammation based on histological verification of the pulmonary pathology: L, low; M, middle; H, high.

[i]Distant, lung tissue far from macroscopic TB lesions and cavities.

[j]The total number of alveolar macrophages produced from lung tissue sample of TB patient.

[k, n]Data are presented as percentage of the number of particular cell type out of the total number of examined macrophages.

[l]The total number of alveolar macrophages with *Mtb* (single or as colonies) produced from lung tissue sample of TB patient.

[m]Data are presented as the percentage of the number of cells with *Mtb* in colonies out of the total number of macrophages with *Mtb*.
